# Crystal Structure of Vaccinia Viral A27 Protein Reveals a Novel Structure Critical for Its Function and Complex Formation with A26 Protein

**DOI:** 10.1371/journal.ppat.1003563

**Published:** 2013-08-22

**Authors:** Tao-Hsin Chang, Shu-Jung Chang, Fu-Lien Hsieh, Tzu-Ping Ko, Cheng-Tse Lin, Meng-Ru Ho, Iren Wang, Shang-Te Danny Hsu, Rey-Ting Guo, Wen Chang, Andrew H. J. Wang

**Affiliations:** 1 Institute of Biological Chemistry, Academia Sinica, Taipei, Taiwan; 2 Department of Life Sciences and Institute of Genome Sciences, National Yang-Ming University, Taipei, Taiwan; 3 Institute of Molecular Biology, Academia Sinica, Taipei, Taiwan; 4 Institute of Biochemical Sciences, National Taiwan University, Taipei, Taiwan; 5 Core Facilities for Protein Structural Analysis, Academia Sinica, Taipei, Taiwan; Washington University, United States of America

## Abstract

Vaccinia virus envelope protein A27 has multiple functions and is conserved in the *Orthopoxvirus* genus of the poxvirus family. A27 protein binds to cell surface heparan sulfate, provides an anchor for A26 protein packaging into mature virions, and is essential for egress of mature virus (MV) from infected cells. Here, we crystallized and determined the structure of a truncated form of A27 containing amino acids 21–84, C71/72A (tA27) at 2.2 Å resolution. tA27 protein uses the N-terminal region interface (NTR) to form an unexpected trimeric assembly as the basic unit, which contains two parallel α-helices and one unusual antiparallel α-helix; in a serpentine way, two trimers stack with each other to form a hexamer using the C-terminal region interface (CTR). Recombinant tA27 protein forms oligomers in a concentration-dependent manner *in vitro* in gel filtration. Analytical ultracentrifugation and multi-angle light scattering revealed that tA27 dimerized in solution and that Leu47, Leu51, and Leu54 at the NTR and Ile68, Asn75, and Leu82 at the CTR are responsible for tA27 self-assembly *in vitro*. Finally, we constructed recombinant vaccinia viruses expressing full length mutant A27 protein defective in either NTR, CTR, or both interactions; the results demonstrated that wild type A27 dimer/trimer formation was impaired in NTR and CTR mutant viruses, resulting in small plaques that are defective in MV egress. Furthermore, the ability of A27 protein to form disulfide-linked protein complexes with A26 protein was partially or completely interrupted by NTR and CTR mutations, resulting in mature virion progeny with increased plasma membrane fusion activity upon cell entry. Together, these results demonstrate that A27 protein trimer structure is critical for MV egress and membrane fusion modulation. Because A27 is a neutralizing target, structural information will aid the development of inhibitors to block A27 self-assembly or complex formation against vaccinia virus infection.

## Author Summary

Mature vaccinia virus has more than 20 envelope proteins, including the A27 protein, which has multiple functions in the virus life cycle. During virus entry, A27 mediates the attachment of mature vaccinia virus to cell surface heparan sulfate. A27 also tethers a viral fusion suppressor protein, A26, to mature virions. During virion morphogenesis, A27 mediates mature virus transport in infected cells. We used X-ray crystallography to determine the structure of tA27 protein, which forms a novel hexamer consisting of four parallel strands and two anti-parallel strands. Hexamerization depends on the coiled-coiled domain from L47 to L82 within each tA27 strand, and mutational analysis revealed that amino acid residues within the coiled-coiled domain are critical for tA27 self-assembly *in vitro*. We extended the importance of tA27 self-assembly into an *in vivo* system in which A27 protein dimer/trimer formation through the coiled-coiled domain is crucial to its biological activity, and revealed how A27 regulates virus-induced membrane fusion through its ability to form complexes with A26 protein. Since A27 is a critical target of neutralizing antibodies against pathogenic poxvirus infection in humans, our findings provide a structural basis for the development of anti-pox drugs.

## Introduction

Vaccinia virus, the prototypic member of the *Orthopoxvirus* genus of the family Poxviridae, contains a double-stranded DNA genome of approximately 190 kb that encodes more than 200 individual proteins [1]. It replicates and produces mature virus (MV) in the cytoplasm of host cells [2]. The vaccinia MV particle contains ∼20 envelope proteins, at least 16 of which participate in MV entry into cells [3], [4]. Three proteins, H3, D8, and A27, mediate MV attachment to the cell surface glycosaminoglycans (GAGs); one A26 protein binds to the extracellular matrix protein laminin [5], [6], [7], [8]. A27 protein was also implicated as a viral fusion protein because a monoclonal antibody recognizing A27 protein neutralized virus entry and interfered with MV-induced membrane fusion [9], [10], [11]. It was proposed that the N-terminal sequences of A27 protein contain hydrophobic residues common to viral fusion peptides and that A27 protein forms parallel trimeric coiled coils common to type 1 fusion proteins [12], [13]. Furthermore, Kochan et al. demonstrated that co-expression of vaccinia A17 and A27 proteins in mammalian and insect cells triggered cell-cell fusion [14], suggesting that A27 protein acts directly in membrane fusion execution. However, 12 additional MV proteins (A16, A21, A28, G3, G9, H2, I2, J5, L1, L5, F9, and O3) were shown to form a viral entry fusion complex (EFC) to mediate membrane fusion, although the fusion mechanism remains unknown [15]. Given the complexity of virion structure, vaccinia virus membrane fusion has been a somewhat contentious issue, and how A27 protein is involved in membrane fusion has been a matter of some debate.

With such a large number of envelope proteins, it is not surprising that vaccinia virus has a wide range of infectivity. Depending on cell types and virus strains, MV particles enter cells through either endocytosis or plasma membrane fusion [16], [17], [18]. Endocytosis of the vaccinia virus WR strain into HeLa cells requires the viral A25–A26 protein complex and two cell surface receptors: integrin β1 [19] and CD98 [20]. The A26 open reading frame (ORF) was deleted from the WR virus genome, and the resulting WRΔA26L mutant virus enters cells through plasma membrane fusion [17], [18]. The current model states that viral A26 protein on MV acts as an acid-sensitive membrane fusion suppressor that binds to the A16 and G9 subcomponents of the EFC to restrain fusion activity at neutral pH [21]. After endocytic uptake of MV into vesicles, the acidic endocytic environment induces the dissociation of A26 protein from MV, leading to viral membrane fusion with vesicular membranes. On the other hand, vaccinia MV lacking A25–A26 suppressor proteins bypass the need for low pH and readily fuse with plasma membrane [17]. While A25 and A26 proteins are important determinants in the vaccinia virus entry process, they are not integral membrane proteins. Therefore, the assembly of A25 and A26 proteins into MV requires A27, which forms disulfide bonds with A26 [22], [23]. Although A27 protein also lacks a transmembrane region, it does interact with the integral membrane protein A17 [24], providing a bridging function to anchor A25 and A26 proteins onto MV particles.

Aside from the role in virus entry described above, A27 protein also facilitates enveloped virus release during the late phase of the viral life cycle. A proportion of MV progeny in infected cells are transported out of viral factories via microtubules through a A27-dependent mechanism to the trans Golgi network, where additional membranes are wrapped and extracellular enveloped virus (EV) is released via exocytosis [25]. Deletion of the A27L gene from the vaccinia virus genome did not affect MV production; however, defects in MV transport [25] and inability to wrap additional membranes [26] were reported. Thus, inactivation of A27 protein functions resulted in the attenuation of EV formation and a small plaque phenotype [27], [28].

Although vaccinia A27 is multi-functional in the vaccinia virus life cycle, its structure is unavailable. We determined the crystal structure of tA27 protein, which reveals unexpected and novel coiled-coil architecture. The availability of the tA27 protein structure allows us to further investigate the oligomeric states of A27 protein and elucidate how it interacts with A26 to regulate membrane fusion during vaccinia virus infection.

## Results

### Structure determination of A27 protein

**Figure 1:**
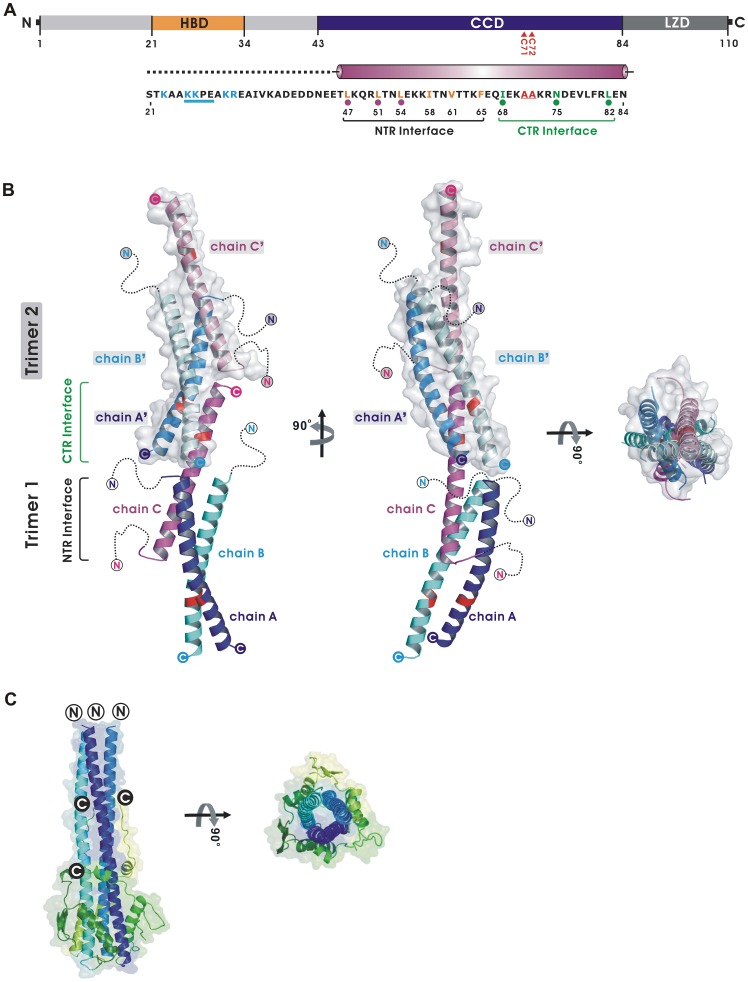
Overall structure of tA27. (A) A schematic representation of the vaccinia virus A27 domain structure. A27 comprises three domains, including the heparin binding domain (HBD), the coiled-coil domain (CCD) for hexameric assembly and interacting with A26, and the leucine zipper domain (LZD) for binding with A17. Expressed tA27 for crystallization studies contains residues from 21 to 84, with mutations in two adjacent cysteine residues (C71A and C72A). The magenta cylinder denotes the region of tA27 structure that corresponds to the α-helix. Black dashed lines indicate the disordered regions. The basic residues in HBD (*cyan*) and the critical segment for specific heparin binding (*underlined*) are shown. The residues in the N-terminal region (NTR) interface involved in trimer assembly are presented in blue, and residues in the C-terminal region (CTR) interface involved in hexamer assembly are in green. Purple and green dots denote the mutation sites for *in vitro* and *in vivo* studies. (B) This representation of hexameric tA27 is composed of trimer 1 (bottom), shown as ribbon diagram and surface model in blue (chain A), cyan (chain B), and magenta (chain C); and trimer 2 (top), presented as surface model and ribbon diagram in light blue (chain A′), light cyan (chain B′), and light magenta (chain C′). The disordered region, including the HBD, is indicated by black dotted lines. Residues 71 and 72 are colored in red. The filled circles indicate the C-terminus and the white circles denote the N-terminus of tA27. (C) The trimeric structure of Influenza virus HA2. A representative trimer-of-hairpins structure of the viral fusion protein of the influenza virus (Flu) HA2 (PDB code 1HTM) is shown [60]. For Flu HA2, the interior N-terminal coiled-coil structures are colored in similar blue and the exterior C-terminal antiparallel helices are in similar green. The right part of the figure shows the model rotated 90°.

Vaccinia A27 protein is composed of 110 amino acid residues containing a heparin binding domain (HBD), a coiled-coil domain (CCD) and the A17 binding leucine zipper domain (LZD) (Figure 1A). The HBD, amino acids (aa) 21–34, includes the core sequence KKPE (aa 26–29), which is structurally flexible and essential for binding to cell surface heparan sulfate (HS) [5], [29], [30]. The CCD contains aa 43–84 and is required for self-oligomerization *in vitro* [29], [31]. The CCD domain contains cysteine 71 and 72, previously found to form disulfide bonds during A27 self-assembly and with A26 protein [23]. The LZD domain (aa 85–110) is the A17 binding region and was predicted to be a leucine zipper [13], [24]. We also expressed a truncated A27 protein (aa 21–84, C71/72A) with a T7 tag at the N-terminus and a hexahistidine tag at the C-terminus, named as T7-tA27, in bacteria with cysteine-to-alanine mutations of both amino acids 71 and 72 to prevent protein insolubility caused by nonspecific disulfide bond formation [31]. C71/72A double mutations had no effect on T7-tA27 protein oligomerization *in vitro* compared with wild type protein, suggesting that disulfide bonding formed between C71 and C72 in the virus-infected cells serves to stabilize A27 oligomer interactions mediated through hydrophobic interactions in the coiled-coil region [13]. The tagged recombinant T7-tA27 protein was soluble and biologically active. It forms hexamers in solution according to a size exclusion chromatography (SEC) study [29], [31], binds to cell surface HS [30], [32], and competes with vaccinia MV for cell attachment [30], [31], suggesting that the T7-tA27 protein mimics the native A27 protein structure on vaccinia MV. Hence, we further purified the tag-free A27 protein (aa 21–84, C71/72A, named as tA27, Figure S1) for X-ray crystallographic study.

The tA27 protein was crystallized in the *P*4_3_2_1_2 space group, and a native dataset was collected to 2.2 Å (Table S1). For phasing determination of the tA27 protein, we also generated a series of tA27 mutants by introducing a methionine residue into the tA27 protein and subsequently producing selenomethionine-labeled crystals of tA27 protein; however, all attempts to produce crystals were unsuccessful. Finally, the initial phases of tA27 protein were solved using multiple-wavelength anomalous diffraction analysis of tantalum bromide cluster derivative crystals (Table 1; Figure S2). The native structure of tA27 protein was refined to an *R*_work_ of 21.0% and *R*_free_ of 27.2%, with all residues located in the allowed regions of a Ramachandran plot (Table 1). The electron density map is shown in Figure S3.

**Table 1:** Data collection and refinement statistics.

	Native	[Ta_6_Br_12_]^+2^	
		Peak	Remote
***Data Collection***			
Source	Spring-8 12B2	NSRRC 13B	NSRRC 13B
ÅWavelength ()	1.00000	1.25516	1.25450
Space group	*P*4_3_2_1_2	*P*4_3_2_1_2	*P*4_3_2_1_2
ÅUnit cell dimensions ()			
*a*	79.19	79.03	79.13
*b*	79.19	79.03	79.13
*c*	90.22	90.26	90.33
ÅResolution ()^a^	30.00 - 2.20 (2.28 - 2.20)	30.00 - 2.62 (2.71 - 2.62)	30.00 - 2.61 (2.70 - 2.61)
No. of unique reflections^a^	15074 (1469)	9074 (892)	9164 (836)
Redundancy^a^	9.3 (9.5)	18.1 (18.5)	9.0 (9.1)
%Completeness ()^a^	99.3 (100.0)	99.9 (100.0)	99.4 (94.7)
σAverage I/(I)^a^	46.2 (5.5)	33.3 (5.3)	30.5 (3.5)
*R*_merge_% ()^a^^,^ b	5.1 (55.5)	7.7 (64.3)	6.0 (63.8)
***Refinement***			
No. of reflections^c^	14299		
*R*_work_/*R*_free_% ()^d^^, ^ e	21.0/27.2		
r.m.s deviations^f^			
Åbond lengths ()	0.018		
°bond angles ()	1.652		
*B*Å-value (^2^)/No. atoms			
Protein	29.3/1036		
Ligand	73.2/63		
Water	62.7/131		
%Ramachandran plot ()			
Most favored regions	98.3		
Allowed regions	1.7		
Disallowed regions	0.0		

a Values in parentheses correspond to the highest resolution data shell.

b *R*_ = merge_∑∑*I*(*h*)*_j_*−< *I*(*h*>∑∑)/ *I*(*h*)*_j_* where *I*(*h*)*_j_* is the measured diffraction intensity and the summation includes all observations.

c All positive reflections were used in the refinement.

d *R*_work is_ = ∑| the R-factor(*F_o_*|−∑| *F_c_*|∑|)/*F_o_*| where *F_o_* and *F_c_* are observed and calculated structure factors, respectively.

e *R*_free_% is the R-factor calculated using 5 of the data that were excluded from the refinement.

f The r.m.s deviation is the root-mean-square deviation from ideal geometry of protein.

### Overall structure of tA27 protein

Monomers composed of tA27 protein fold into single α-helix structures and assemble further into a hexameric structure using a trimer (which corresponds to the crystallographic asymmetric unit) as a building block (Figure 1B). The trimeric building block of tA27 contains unusual triple-stranded coiled-coil interactions with two parallel α-helices (chains A and B) and one antiparallel α-helical strand (chain C) using the N-terminal region interface (NTR Figure 1B). The antiparallel α-helix (chain C) uses its C-terminal region interface (CTR) to form a bridge-like structure that connects two trimers into a hexameric assembly. This unique type of parallel-to-antiparallel interaction within each trimer is distinct from the classical parallel trimeric coiled-coil structure common to other viral fusion proteins, such as influenza virus HA2 (Figure 1C; Table S1), although the individual coiled-coil of tA27 and the heptad repeat of viral fusion proteins are structurally similar. Each subunit of tA27 has conformational differences, with average root mean square deviations of 2.71 Å (chains A and B; 40 Cα atoms), 2.08 Å (chains A and C; 40 Cα atoms), and 1.28 Å (chains B and C; 40 Cα atoms) (Figure S4). The hexameric assembly of tA27 is a structurally stable unit with the NTR and CTR providing significant driving force for protein oligomerization. This finding is supported by the Protein Interfaces, Surface, and Assemblies (PISA) program and our previous CD spectroscopy analyses (Table S2) [29], [33]. Previous reports also demonstrated that both full length and T7-tA27 form trimers/hexamers *in vitro* and that oligomerization of A27 protein is concentration-dependent [13], [29], [30], [31].

The HBD of tA27 is a short peptide region of aa 21–34 [32]. Using site-directed mutagenesis and solution NMR, we previously identified four amino acids, KKPE, with a turn-like conformation that mediates specific heparin binding [30]. The HBD feature is distinct from the foot-and-mouth disease virus, which contains a shallow cavity for binding to HS on cells, and the adeno-associated virus, whose capsid proteins form a channel-like structure to interact with heparin [34], [35], [36], [37]. Due to the intrinsic flexibility of HBD [29], its electron density maps (residues 21–44 for chains A and B; residues 21–43 for chain C) were not visible (Figure 1B; Figure S3). Despite extensive efforts to co-crystallize tA27 proteins and soak native tA27 crystals with HS and heparin, we have not obtained the ligand-bound form of tA27 structures. It might be that the structure of the HBD is only induced upon binding to its ligand and that ligand association and dissociation are in a dynamic equilibrium. In the future, it will be worthwhile to investigate tA27 protein in complex with heparin or HS using small-angle X-ray scattering.

**Figure 2:**
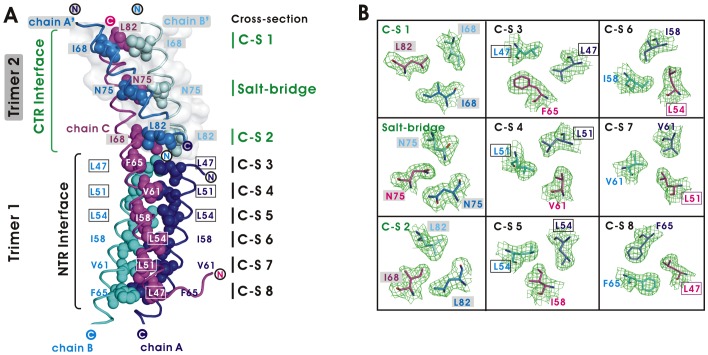
Interactions of the N-terminal region (NTR) and the C-terminal region (CTR) interfaces. (A) The residues in the CTR interface are depicted as sphere models (chain A′, *light blue; *′ chain B′, *light cyan*). They are divided into two layers of cross-sections (C-S) for hydrophobic interaction and one hydrogen-bonding interaction. The hydrophobic resides involved in the NTR interface are shown as a cluster of spheres in blue (chain A), cyan (chain B), and magenta (chain C), and divided into 8 layers of C-S. The filled circles denote the C-terminus and the open circles indicate the N-terminus of tA27. The triple L-to-A mutation sites at the NTR interface are boxed. (B) The 2| *F*_O_|-|*F*_C_| electron density maps are contoured at 1.0σ level as green mesh, and the refined protein model is shown as sticks in blue (chain A′), cyan (chain B′), magenta (chain C), light blue (chain A′), and light cyan (chain B′).

The CCD of tA27 protein contains approximately 42 residues (aa 43–84). In the CCD region, six hydrophobic residues, including Leu47, Leu51, Leu54, Ile58, Val61, and Phe65, are packed in six layers at the NTR, along with Ile68, Asn75, and Leu82 at the CTR (Figure 2). These residues are located in close proximity with one another, and along with the hydrogen bonds of Asn75 they mediate hydrophobic interaction as the primary driving force for the molecular self-assembly of the tA27 protein (Figure 2B; Table S2. Specifically, Cross-Section 1 (C-S 1) is composed of Ile68 (chains A′ and B′) and Leu82 (chain C) C-S 2 contains Leu82 (chains A′ and B′) and Ile68 (chain C); C-S 3 contains Leu47 (chains A and B) and Phe65 (chain C); C-S 4 includes Leu51 (chains A and B) and Val61 (chain C); C-S 5 contains Leu54 (chains A and B) and Ile58 (chain C); C-S 6 is composed of Ile58 (chains A and B) and Leu54 (chain C); C-S 7 comprises Val61 (chains A and B) and Leu51 (chain C); and C-S 8 consists of Phe65 (chains A and B) and Leu47 (chain C). Such parallel-to-antiparallel multiple-strand arrangements of hydrophobic residues provide a strong structural basis to explain the previous *in vitro* study that demonstrated triple mutations (TM) of L47A, L51A, and L54A, which resulted in T7-tA27 disassembly [31] because all six hydrophobic interactions (shown in Figure 2) are completely disrupted. These arrangements of hydrophobic interaction also solved a previous disagreement concerning a proposed model [13] that predicts three parallel coiled-coil structures of A27 trimer, in which three bulky face-to-face Phe65 residues would have disrupted A27 trimer assembly. In fact, it is critical to point out that the three coiled-coil arrangements in the tA27 crystal structure allow spatial stability of the A27 assembly. In addition, the Asn75 side chain OD1 of chain C forms two hydrogen bonds with the Asn75 NH2 of chains A′ and B′. Most importantly, the tA27 crystal structures are in agreement with the biochemical properties of soluble T7-tA27 protein reported previously (see below).

Previous studies showed that both HBD and oligomerization of T7-tA27 protein are required for cell attachment [30], [31], [38]. The conclusion was based on experiments showing that a 14-mer peptide containing only the HBD exhibited minimal affinity to heparin/HS [30] and that *in vitro* leucine-to-alanine mutagenesis of L47, L51, and L54 (triple mutations, TM) of T7-tA27 protein generated a soluble T7-tA27-TM protein that failed to bind to heparin *in vitro* and to HS on cells [31]. The NMR spectra for T7-tA27 protein revealed slow molecular dynamics due to protein self-assembly [29], and CD spectroscopy analysis showed that TM destabilized T7-tA27 protein into monomeric subunits at near-neutral pH (∼6.7) [31].

In addition to self-assembly through the CCD region, it is worth noting that A27 protein also associates with other viral proteins. The two cysteine 71/72 residues, although mutated to alanine in the tA27 crystal structure (marked red in Figure 1), mediate intermolecular disulfide bonds with Cys441 and Cys442 of the A26 protein [23]–. Two forms of A26A27 protein complexes (70-kDa and 90-kDa) were detected on MV particles in non-reducing conditions [21], [23]. The C-terminal LZD region of the A27 protein, although absent from the tA27 crystal structure, reportedly interacts with viral integral protein A17 to facilitate A27 anchoring onto MV particles [13], [14], [24].

**Figure 3:**
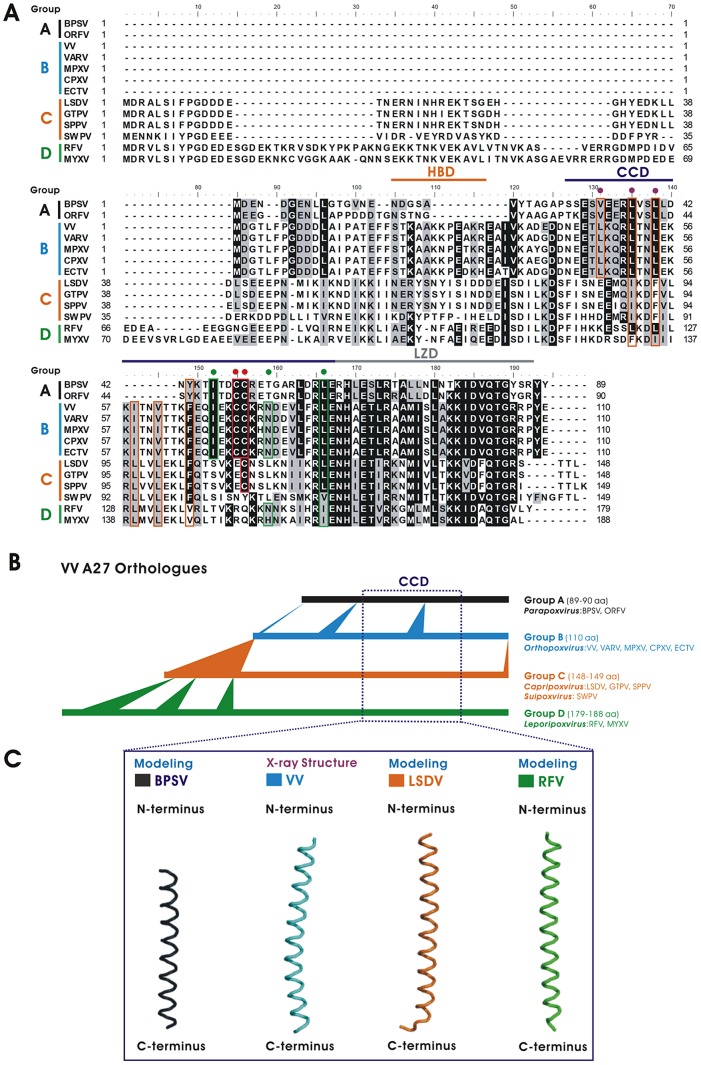
Analysis of vaccinia A27 orthologs. (A) Multiple sequence alignment of A27 orthologs. The alignment contains group A from Parapoxvirus, including Bovine papular stomatitis virus (BPSV) and Orf virus (ORFV) group B from Orthopoxvirus including Vaccinia virus (VV), Variola virus (VARV), Monkeypox virus (MPXV), Cowpox virus (CPXV), and Ectromelia virus (ECTV) group C from Capripoxvirus, including Lumpy skin disease virus (LSDV), Goatpox virus (GTPV), Sheeppox virus (SPPV), and Suipoxvirus with Swinepox virus (SWPV); and group D from Leporipoxvirus, including Rabbit fibroma virus (RFV) and Myxoma virus (MYXV). Note the heparin binding domain (HBD, *orange*), coiled-coil domain (CCD, *purple*), and leucine zipper domain (LZD, *grey*) regions. The conserved residues surrounding the CCD are marked with orange boxes for NTR and green boxes for CTR. The two cysteine residues in the CCD are denoted by red boxes and dots. Identical and similar amino acid residues are shaded in black and gray, respectively. Dashes denote the sequence gaps introduced to optimize the amino acid sequence alignment. The accession numbers are: BPSV (NP958013); ORFV (AAR98199); VV (YP233032); VARV (ABF23908); MPXV (NP536566); ECTV (NP671648); CPXV (NP619946); LSDV (NP150551); GTPV (ABS72324); SPPV (NP659689); SWPV (NP570274); RFV (NP052004); and MYXV (NP051829). (B) The protein evolution of A27 orthologs. The respective A27 protein lengths of Groups A, B, C, and D are represented by black, cyan, orange and green lines, respectively. The inserted residues are marked with triangles, and the corresponding conserved regions of CCD are boxed in red dashes. (C) Computer modeling of vaccinia A27 orthologues with the X-ray structures of vaccinia virus tA27 show that the CCD regions are structurally conserved, as predicted by molecular modeling, in group A (BPSV, *black*), group C (LSDV, *orange*), and group D (RFV, *green*). The predicted modeling structures were produced by the Phyre server [61].

Vaccinia A27 protein orthologs are widely conserved in Poxviridae (Figure 3A). Alignment of poxviral A27 orthologs reveals that the CCD and LZD domains are more conserved than the HBD domain among parapoxvirus (Group A), orthopoxvirus (Group B), capripoxvirus, suipoxvirus (Group C), and leporipoxvirus (Group D). The different sizes of A27 ortholog proteins in each group mainly reflect deletions/insertions clustered at the N-terminal region of A27 orthologs, while the domain sizes of the CCD and LZD are relatively conserved (Figure 3B). For the CCD, the hydrophobic residues contributing to the formation of the coiled-coil structure are particularly conserved in Groups B, C, and D (Figure 3A). In addition, cysteine residues within the CCD of the vaccinia A27 protein are conserved in Groups A, B, and C. Threading the sequences of A27 orthologs onto the X-ray structure of vaccinia tA27 protein for structure modeling reveals that the CCD domains of A27 orthologs exhibit conserved structural folding (Figure 3C), implying that these A27 orthologs evolve to form similar structural architecture in order to maintain important biological functions.

### Size exclusion chromatography (SEC) of wild type and mutant tA27 proteins

**Figure 4:**
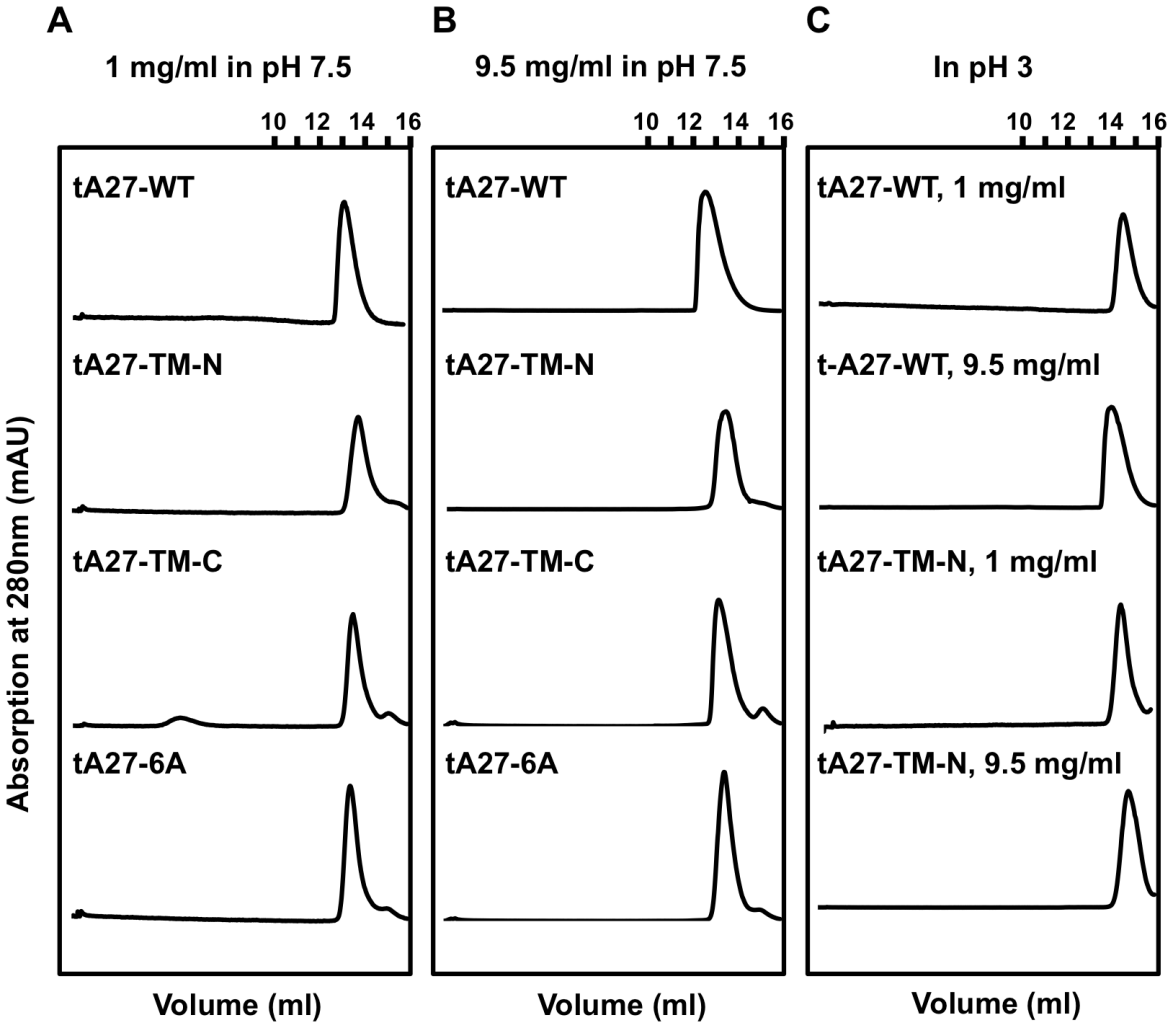
Size exclusion chromatography of tA27-WT protein showing oligomer formation in a concentration-dependent manner. (A and B) Recombinant tA27-WT, tA27-TM-N, tA27-TM-C, and tA27-6A proteins at concentrations of 1 mg/ml (A) and 9.5 mg/ml (B) were individually injected into a Superdex 75 10/300 GL size exclusion column at pH 7.5, and the protein elution profiles at 280 nm UV absorption were recorded. The apparent molecular mass of each tA27 recombinant protein was determined using standard protein molecular mass markers (Table S3). (C) tA27-WT and tA27-TM-N proteins at concentrations of 1 mg/ml and 9.5 mg/ml were analyzed using the same size exclusion system equilibrated at pH 3.0. Then, the protein elution profiles were recorded. Because protein markers were not suitable for use as standards at low pH, the protein elution volume was used to compare different recombinant proteins.

A previous *in vitro* study demonstrated that TM of L47A, L51A, and L54A resulted in T7-tA27 disassembly [31], which showed the important contribution of residues at the NTR to T7-tA27 protein assembly however, the importance of residues in the CTR was not investigated. Therefore, we generated a series of tA27 expression constructs to re-visit these issues. The mutation sites in tA27 protein (Table S3) include tA27-WT containing C71/72A (tA27-WT); tA27-TM, L47A/L51A/L54A, at the N-terminal interface (tA27-TM-N); tA27-TM, I68A/N75A/L82A, at the C-terminal interface (tA27-TM-C); and tA27-hexa alanine mutations combining both L47A/L51A/L54A and I68A/N75A/L82A (tA27-6A). Because the stretch of tA27 protein encompassing amino acids 21–84 does not contain any aromatic residues, an extra tryptophan was included at the N-terminus of each protein. All of the recombinant proteins were purified as tag-free tA27 proteins, as described in materials and methods. Each recombinant protein of 1 mg/ml and 9.5 mg/ml was individually injected into a Superdex 75 10/300 GL size exclusion column at pH 7.5, the fractions were collected, and the molecular mass of each tA27 recombinant protein was determined using standard protein molecular mass markers. Additionally, tA27-WT and tA27-TM-N proteins were analyzed by gel filtration system equilibrated at pH 3.0 (which has been reported to induce dissociation of tA27-WT protein into monomers by 2D NMR experiments [31]). They were subsequently analyzed using a gel filtration system equilibrated in pH 3.0, and fractions were collected. Because protein markers were unsuitable as standards at low pH, the protein elution volume was used for comparison among different recombinant proteins. The SEC results are summarized in Figure 4 and Table S3. At 1 mg/ml low concentration, all four proteins (tA27-WT, tA27-TM-N, tA27-TM-C, and tA27-6A) were eluted with a very close peak volume with respective apparent molecular masses of 24.4, 19.8, 21.9, and 22.9 kDa, suggesting these proteins were trimers. At 9.5 mg/ml, tA27-WT was eluted earlier, suggesting concentration-dependent oligomerization, while the elution volumes of tA27-TM-N, tA27-TM-C, and tA27-6A remained largely unchanged. Strangely, when tA27-WT and tA27-TM-N at 1 mg/ml were eluted at pH 3.0, at which both proteins should exist mostly as monomers, the elution volumes remained largely unchanged. The obvious discrepancy between the apparent molecular mass and elution volume of the monomers raised the possibility that tA27 protein, with an elongated asymmetric shape, is not suited for use in SEC for oligomer determination since most molecular standard proteins are globular in shape. Indeed, the length and diameter of the tA27 helix might disguise the protein, causing it to behave like a much larger protein in solution because its radius of gyration would be larger owing to its asymmetric shape.

### Analytical ultracentrifugation and multi-angle light scattering analyses of tA27-WT and mutant proteins

Next, we sought to use two independent biophysical methods, sedimentation velocity analytical ultracentrifugation (AUC-SV) and size exclusion chromatography with multi-angle light scattering (SEC/MALS) to elucidate the oligomeric state of recombinant tA27-WT in solution. Both methods can be used to determine the molecular weights of proteins without assuming that the protein of interest is compact and globular [39].

**Figure 5:**
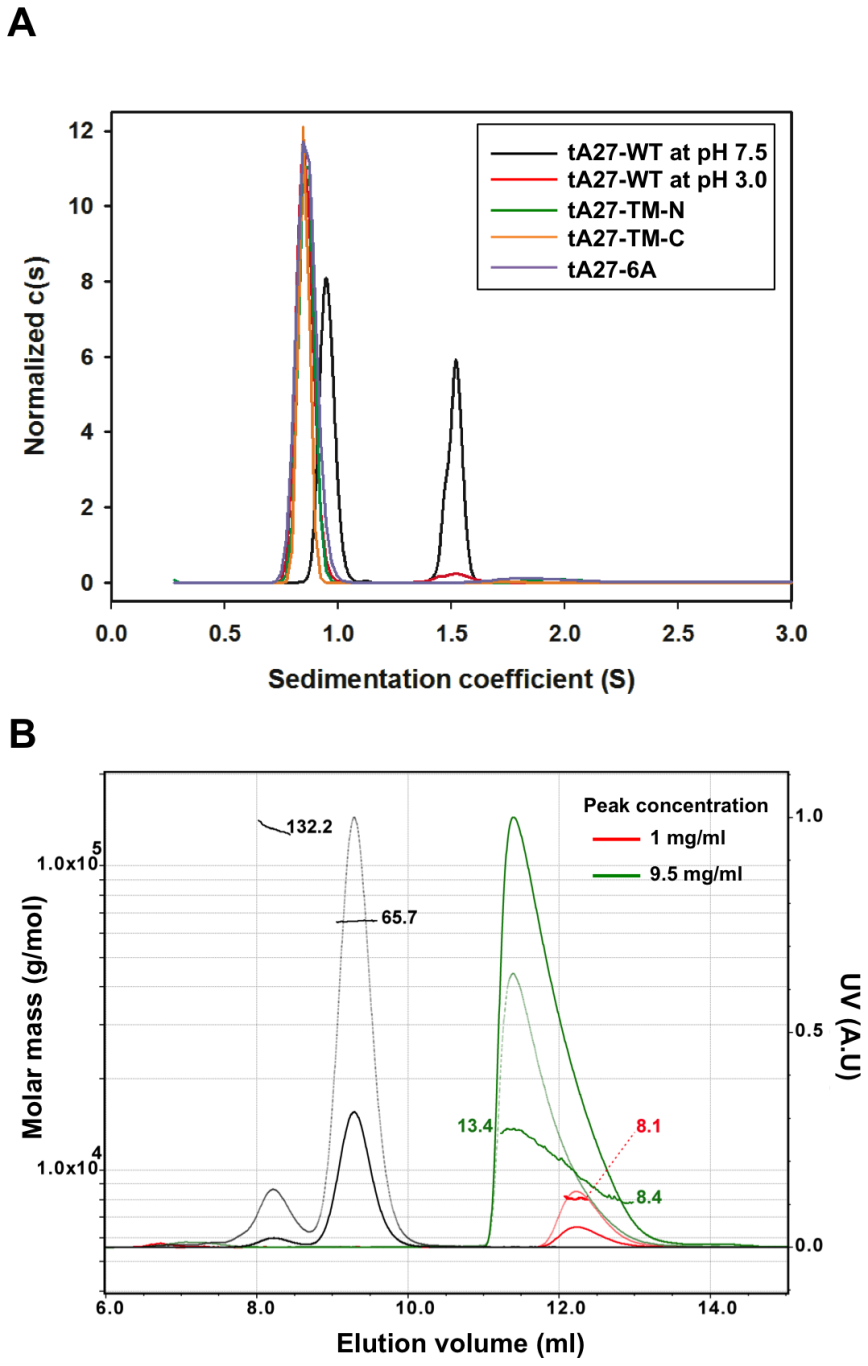
AUC-SV and SEC/MALS analysis of tA27 protein showing that tA27 protein formed concentration-dependent dimers. (A) AUC analyses of tA27-WT protein. Normalized *c*(*s*) distribution plots for tA27-WT and mutants. The figure shows the distribution plots for tA27-WT at pH 7.5 (*black*), tA27-TM-N (*green*), tA27-TM-C (*orange*), tA27-6A (*purple*) and tA27-WT at pH 3.0 (*red*) obtained from the fitting of SV data using a continuous *c*(*s*) distribution model. (B) SEC/MALS profiles of tA27-WT protein at concentrations of 1 mg/ml (*red*) and 9.5 mg/ml (*green*). BSA was used as control (*black*). Thin line segments represent the calculated molar masses; the numbers denote the corresponding molecular weights of each peak (left ordinate axis) in kDa. Solid lines represent normalized UV absorbance (280 nm, right ordinate axis), and dashed lines represent light scattering.

tA27-WT, tA27-TM-N, tA27-TM-C, and tA27-6A, each at 120 µM concentration, were examined in solution by AUC-SV as described in the materials and methods. The results of c(s) analysis for tA27-WT at pH 7.5 showed two different peaks (0.96S and 1.5S) suggesting concentration-dependent oligomerization (Figure 5A). The small peak at ∼0.96 S is assigned to the monomeric form, while the peak at 1.5S corresponds to a molecular mass of ∼16 kDa and is assigned to the dimeric form. tA27-WT at pH 3.0 and tA27 mutants TM-N, TM-C, and 6A at pH 7.5 all remained as a major peak at 0.88S, indicating monomeric structure. The small difference of the tA27-WT monomers sedimentation coefficient at neutral and acidic pH (0.96S vs. 0.88S) may be attributed to other factors, such as an acidic environment affecting the hydrodynamic parameters or shape and integrity of the structure of tA27-WT. The SV result of tA27-WT at a low concentration (20 µM) has only a broad peak in the range of 0.75S to 1.1S and lacks a significant peak around 1.5S (data not shown), indicating that the proportions of monomer and oligomers changed depending on the total protein concentration, with the proportion of dimer apparently increasing as the concentration increased.

We also employed SEC/MALS to determine the absolute molecular weights of tA27-WT moieties in solution based on the angular dependence of scattered light intensity, which is independent of the molecular shapes, thereby circumventing the issue of asymmetric molecular assembly of tA27-WT that contains disordered N-terminal fragments. At a low protein concentration (1 mg/ml), the retention volume (ca. 12 ml) of tA27-WT in our SEC/MALS analysis was consistent with the previously reported value using a Superdex 75 10/300 GL column; however, MALS analysis indicated that the molecular mass of this fraction is 8.1±0.3 kDa, which corresponds to monomeric tA27-WT and indicates that the sample is monodispersed (Figure 5B). At a much higher protein concentration (9.5 mg/ml), the retention volume became smaller (ca. 11 ml) and the corresponding peak was highly asymmetric, indicating that tA27-WT becomes polydispersed in solution with a rapid equilibrium between different oligomeric states. Indeed, the SEC/MALS analysis indicated a continuous molecular mass distribution ranging from 13.4±0.4 kDa to 8.4±0.2 kDa for the asymmetric elution peak. The result suggested that tA27-WT monomer and dimer most likely coexisted at an equilibrium. However, we did not identify the existence of trimeric tA27-WT in solution within this protein concentration range. In conclusion, our SEC/MALS data suggested that tA27-WT protein exists primarily as a monomer in solution at low protein concentration, and becomes more dimeric as its concentration increases. While the results may seem contradictory to the crystallographic findings, it is important to note that the SEC/MALS and AUC analyses are performed in solution while protein crystallization is by default an oligomerization process, which has a very high local concentration. Although tA27-WT was detected as monomer-dimers in solutions, it is important to point out that protein behavior in solution is fundamentally different from protein crystal environments. As tA27-WT exhibits increased oligomerization propensity at high concentration, it is plausible that trimer/hexamer formation (as observed in the crystal structure) can occur *in vivo* where the local concentration is elevated. Because it was difficult to obtain the soluble form of full-length A27 to use in further experiments, we sought to use genetic approaches to address the structure/function relationship of A27 protein *in vivo*.

### A27 TM-N, TM-C, and 6A mutations interrupted the formation of A27 dimer, A27 trimer, and the A26-A27 70/90-kDa complex in the transient expression system

We performed *in vitro* mutagenesis, introduced TM-N, TM-C, and 6A mutations into the full-length A27 ORF, and cloned into a plasmid pMJ601 [40] such that a viral synthetic late promoter drove A27 protein expression in the infected cells. We then used a transient transfection-infection system to investigate A27 protein self-assembly in non-reducing conditions, since the A27 trimers are stabilized *in vivo* by disulfide bonding [11], [23]. We monitored the 70-kDa and 90-kDa protein complexes that are also formed between A26 and A27 through disulfide bonding [21], [22], [23].

**Figure 6:**
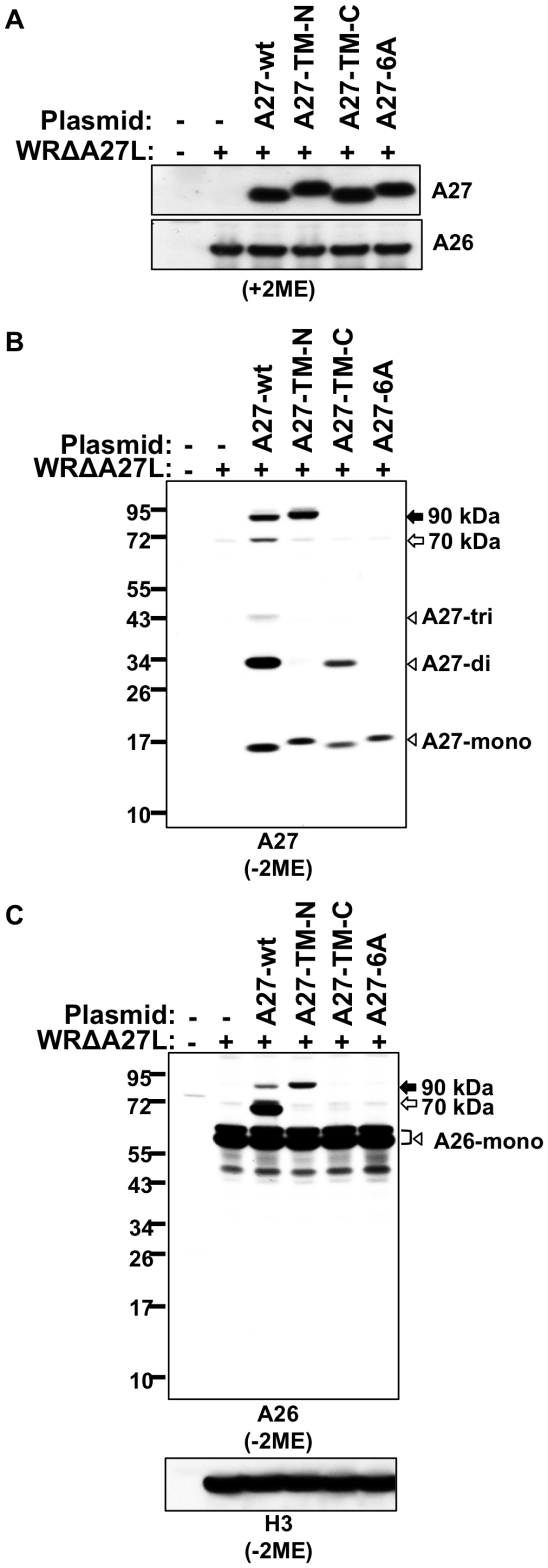
Immunoblot analysis of A27 proteins in transiently transfected-infected cells. 293T cells were infected with WRΔA27L virus and subsequently transfected with plasmids containing A27-wt, A27-TM-N, A27-TM-C, or A27-6A DNA. Lysates were harvested at 24 h post-infection and separated on 4% to 12% SDS-PAGE gels in reducing (+2ME) (A) or non-reducing (−2ME) conditions (B and C) for immunoblot analysis with anti-A27 (1∶1,000) (A & B) and anti-A26 (1∶5,000) (A & C) antibodies. The arrowheads labeled A27-mono, A27-di, and A27-tri represent A27 protein monomer, dimer, and trimer, respectively. The black and white arrows mark the 90-kDa and 70-kDa A26–A27 protein complexes, respectively. A26-containing bands appeared as doublets because of the incomplete formation of intramolecular disulfide bonding between C43 and C342 in the A26 protein [23].

293T cells were transiently transfected with individual A27 plasmid constructs, subsequently infected with WRΔA27L at a multiplicity of infection (MOI) of 5 plaque-forming units (PFU)/cell and harvested at 24 h post-infection for SDS-PAGE analysis. In reducing conditions, comparable levels of each transfected A27 protein construct were detected in cells; the endogenous A26 protein level in WRΔA27L was similar (Figure 6A). Next, we probed a non-reducing gel with A27 antibody (Figure 6B). WT A27 protein formed monomer/dimer/trimer and 70-kDa and 90-kDa A26–A27 protein complexes in cells, while A27-TM-N protein mainly existed as a monomer (with a trace amount of dimer) and as part of the 90-kDa A26–A27 protein complex. A27-TM-C protein was present in monomer and dimer forms, and did not form any complex with A26. Finally, A27-6A protein only existed as a monomer and did not form any complex with A26 protein (Figure 6B). These results suggest that the TM-N and TM-C mutations each affect A27 protein self-assembly to a different extent, and combining all six mutations in A27-6A eliminated its ability to self-assemble.

The TM-N mutation only affected formation of the 70-kDa complex, while the TM-C mutation abolished formation of both the 70-kDa and the 90-kDa A26–A27 complex. The lysates were probed with anti-A26 antibody in non-reducing conditions, and the results are consistent (Figure 6C). H3 protein level in each lane was comparable as stained by anti-H3 antibody (Figure 6C). We want to emphasize that the lack of formed complexes consisting of a combination of A27-TM-C or A27-6A with A26 protein cannot be interpreted as a simple result of insufficient A26 protein level in cells (Figure 6A and C). Each of the A26-containing bands that appeared as doublets were observed before and caused by the incomplete formation of intramolecular disulfide bonds between C43 and C342 in the A26 protein [23].

### A27 protein containing TM-N, TM-C, and 6A mutations failed to mediate MV egress

**Figure 7:**
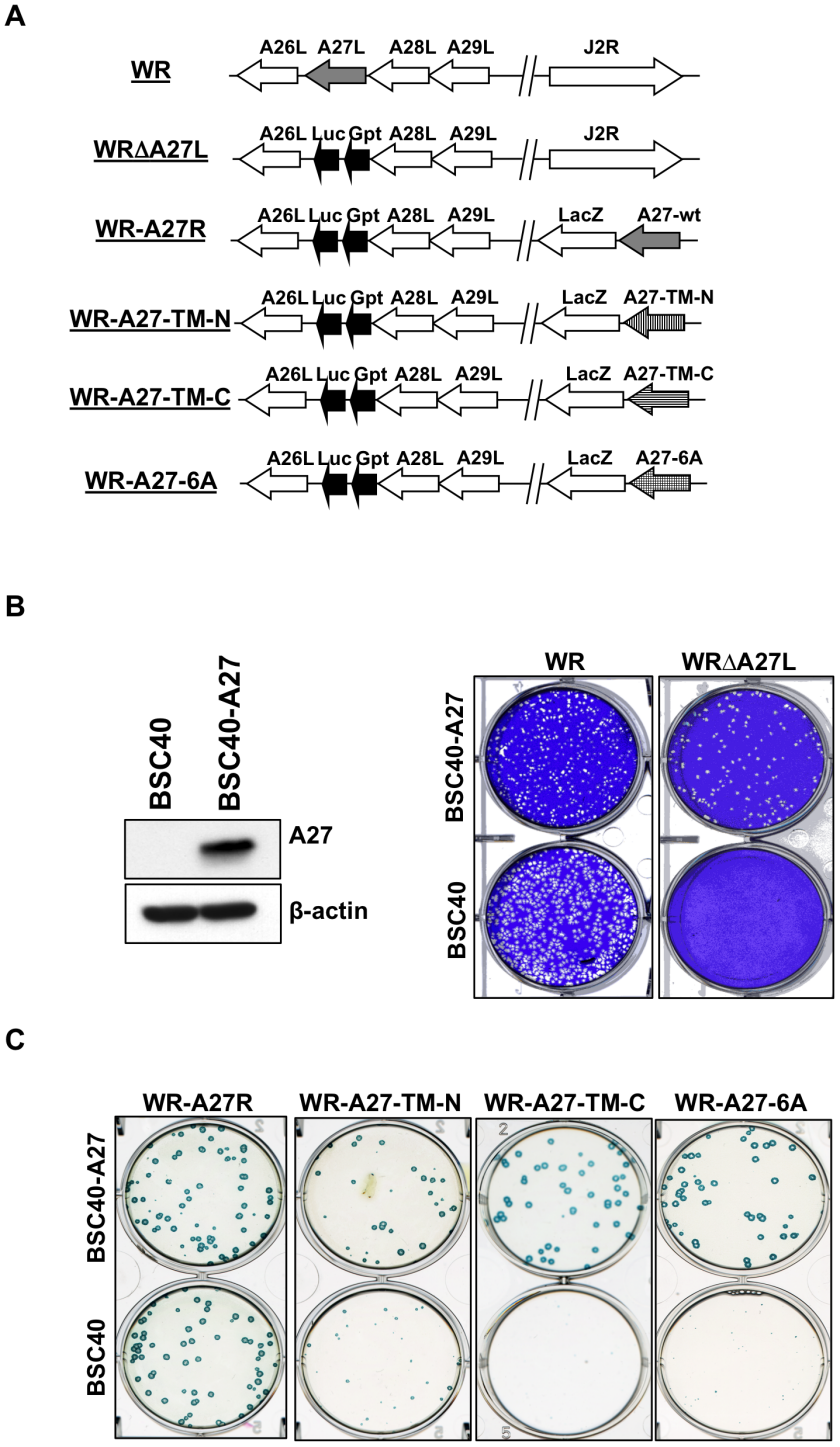
WR-A27-TM-N, WR-A27-TM-C, and WR-A27-6A exhibited a small plaque phenotype on BSC40 cells. (A) Schematic representation of vaccinia virus genomes containing A26–A29 and J2R ORFs. Wild type WR virus is shown at the top, with arrows indicating the direction of transcription. In WRΔA27L, WR-A27R, WR-A27-TM-N, WR-A27-TM-C, and WR-A27-6A, the viral A27L ORF was replaced with a dual expression cassette, Luc-Gpt, containing a luciferase (Luc) gene driven by a viral early promoter and the Eco-gpt (Gpt) gene driven by the viral p7.5 promoter. The J2 locus of the WR-A27R, WR-A27-TM-N, WR-A27-TM-C, and WR-A27-6A genome was inserted with another dual expression cassette containing an A27 ORF driven by a viral late promoter and a *lacZ* gene driven by the viral p7.5k promoter. (B) BSC40-A27 cells express A27 protein for function complementation in *trans*. Immunoblot analysis of A27 protein expressed in BSC40-A27 cells. Beta-actin was used as a control. WRΔA27L virus, which formed small plaques on BSC40 cells, produced large plaques on BSC40-A27 cells after cell staining with 1% crystal violet in 20% ethanol. (C) Virus plaque morphology on BSC40 and BSC40-A27 cells. WR-BSC40-A27 and BSC40 cells were infected with 50–100 plaques of WR-A27R, WR-A27-TM-N, WR-A27-TM-C, and WR-A27-6A, fixed at 2 days post-infection and subsequently stained with X-gal and photographed.

Encouraged by the transient assay results, we constructed recombinant vaccinia viruses expressing A27 mutant proteins (Figure 7AΔ). Removal of the A27 gene from wild type vaccinia virus caused a defect of MV egress resulting in a small plaque phenotype on BSC40 cells, as observed for WRA27L [27], [28]Δ. Therefore, we generated a BSC40-A27 cell line on which even WRA27L viruses grew as large plaques because A27-WT protein is constitutively expressed in *trans* (Figure 7B). A dual expression cassette containing a full-length A27 ORF encoding either A27-WT (to construct a revertant virus, A27R), A27-TM-N, A27-TM-C, or A27-6A mutant protein, and a marker *lacZ* gene was inserted into the J2R (*tk*Δ) locus of WRA27L virus (Figure 7A).

**Figure 8:**
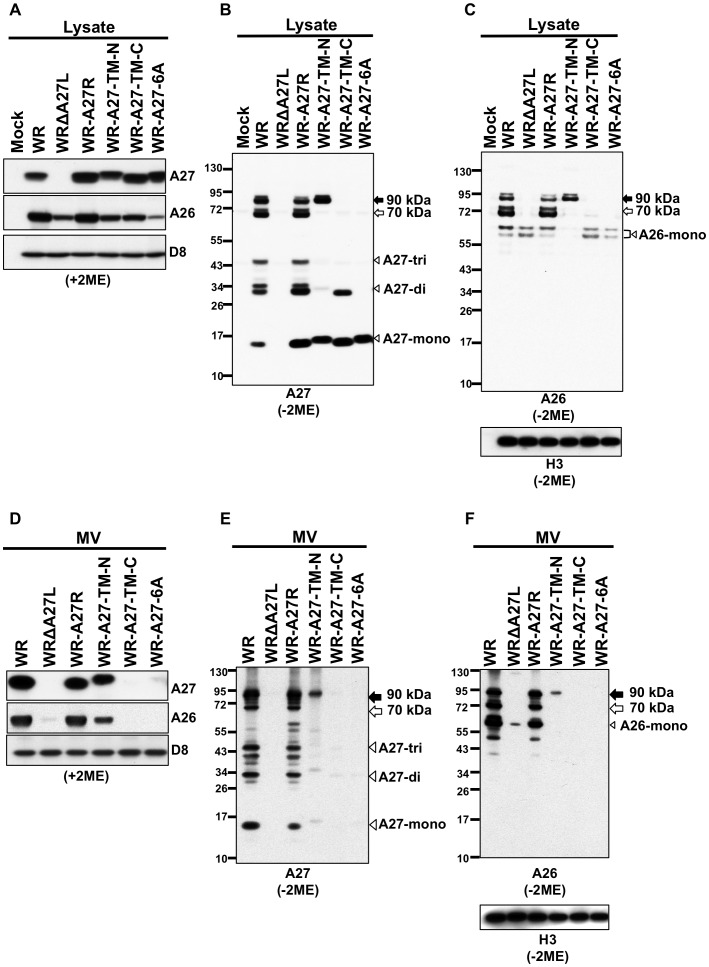
Immunoblot analysis of A27 protein in infected cells and on mature virus particles. (A) HeLa cells were mock-infected or infected with WR, WRΔA27L, WR-A27R, WR-A27-TM-N, WR-A27-TM-C, and WR-A27-6A at an MOI of 5 PFU/cell. Lysates were harvested at 24 h post-infection and separated on 4% to 12% SDS-PAGE gels in reducing conditions (+2ME) for immunoblot analysis with anti-A26 (1∶1,000), anti-A27 (1∶5,000), and anti-D8 (1∶5,000) antibodies. (B and C) The same infections as in A were processed for immunoblot analysis in non-reducing conditions (−ME) and probed with anti-A27 (B) and anti-A26 and anti-H3 (C) antibodies. A26-monomers (A26-mono) appeared as doublets because of incomplete intramolecular disulfide bonding between C43 and C342 of the A26 protein [23]. (D) Purified MV particles (1µg) of WR, WRΔA27L, WR-A27R, WR-A27-TM-N, WR-A27-TM-C, and WR-A27-6A were separated on 4%–12% SDS-PAGE gels and processed for immunoblot analysis in reducing conditions (+2ME) as described above. (E and F) Immunoblot analysis of purified MV in non-reducing conditions (−2ME) using anti-A27 (E) and anti-A26 and anti-H3 (F) antibodies. The black and white arrows mark 90-kDa and 70-kDa A26–A27 protein complexes, respectively. Arrowheads – labeled mono, -di, and -tri represent protein monomer, dimer, and trimer, respectively.

The four resulting recombinant viruses were isolated as large blue plaques by X-gal staining of BSC40-A27 cells (Figure 7C). Although all of the recombinant viruses expressed similar levels of A27 protein in BSC40 cells (Figure 8A), only the WR-A27R virus formed large plaques and WR-A27-TM-N, WR-A27-TM-C, and WR-A27-6A formed small blue plaques on BSC40 cells (Figure 7C). Since A27 protein is necessary to mediate MV egress leading to EV formation and large plaques, the results suggest that mutations in A27-TM-N, A27-TM-C, and A27-6A knocked out A27 protein function to mediate MV transport. It is worth mentioning that only A27 (not A26) is involved in MV egress, as deletion of the A26L ORF did not alter the large plaque phenotype on BSC cells [8]. Consistent with the small plaque phenotype, BSC40 cells infected with WR-A27-TM-N, WR-A27-TM-C, and WR-A27-6A produced less actin-containing EV [25], [26] in immunofluorescence analysis (data not shown). Taken together, we concluded that residues at both N- and C-terminal trimer interfaces of A27 protein are important for mediating MV egress in infected cells.

### A27 TM-N, TM-C, and 6A mutations reduced A26 protein stability in virus-infected cells and the amount of A26 and A27 protein on MV particles

After obtaining all of the recombinant viruses, BSC40 cells were infected with wild type WR, WRΔA27L, WR-A27R, WR-A27-TM-N, WR-A27-TM-C, and WR-A27-6A at an MOI of 5 PFU/cell, and subsequently harvested at 24 h post-infection for SDS-PAGE analysis in reducing (+2ME Figure 8A) or non-reducing (−2ME) conditions with anti-A27 (Figure 8B), anti-A26 and anti-H3 (Figure 8C) antibodies. Similar to what was observed in the transient expression experiment, A27-WT protein in cells infected with WR and WR-A27R revertant viruses self-assembles into A27 monomer/dimer/trimer (Figure 8B). Some A27 dimer existed as doublets, and the reason is not clear. A27L-TM-N and A27-TM-C proteins self-assemble into monomer/dimer although the proportions of monomer and dimer appeared to be different for each protein. Most interestingly, A27-6A protein was only present as a monomer in cells. Taken together, the A27 immunoblot results suggest that both NTR and CTR interfaces contribute to A27 trimerization and simultaneous interruption of NTR and CTR interfaces dissociates A27 protein into monomers in the infected cells, supporting the A27 crystal structure.

We used anti-A26 antibody, to monitor A26 protein level in cells in reducing conditions (Figure 8A). The A26 protein level was very sensitive to the deletion and mutation of A27 protein, not because of nonspecific interruption of A26 transcription or translation, but because WR-A27R revertant virus expresses a high level of A27-WT protein at the same tk locus as these A27 mutant viruses. In the non-reducing condition (Figure 8C;), only WR and WR-A27R contained abundant A26, which formed 70-kDa and 90-kDa complexes in cells. While WR-A27-TM-N, WR-A27-TM-C, and WR-A27-6A all contained less A26 protein, WR-A27-TM-N remained partly able to form the 90-kDa A26-A27 complex WR-A27-TM-C and WR-A27-6A formed neither the 70-kDa nor the 90-kDa complex (Figure 8C). Combining the results of transient assays (Figure 6) and recombinant viruses (Figure 8C), we concluded that the A27 trimer structure is critical for A26 interaction and protein stabilization.

Next, we produced MV particles from the infected cell cultures, purified them using CsCl centrifugation, and subjected them to SDS-PAGE and immunoblot analysis in the reducing (+2ME) condition (Figure 8D). WR and WR-A27R MV particles contained abundant A26 and A27 proteins. WR-A27-TM-N contained reduced but detectable amount of A26 and A27, while WR-A27-TM-C and WR-A27-6A contained little A26 or A27. All of the purified MV particles contained similar amounts of H3 protein (Figure 8F). When MV particles were analyzed in the non-reducing condition (−2ME) with anti-A27 (Figure 8E), anti-A26 and anti-H3 antibodies (Figure 8F), the conclusion was consistent with what was observed in the cell lysates after transient expression (Figure 6) and the virus-infected cell lysates (Figure 8B and C). Together, the results demonstrate that interruption of A27 self-assembly resulted in partial or complete loss of A26 and A27 protein packaging into MV particles.

### MV particles purified from cells infected with WR-A27-TM-N, WR-A27-TM-C, and WR-A27-6A triggered plasma membrane fusion at neutral pH

Our previous study results suggested that vaccinia A26 protein on MV controls the pathway specificity of viral entry into HeLa cells [17]. Wild type WR strain MV particles contain A26 protein and enter HeLa cells through an endocytosis pathway however, WRΔA26L MV particles enter cells through plasma membrane fusion, resulting in robust cell-cell fusion at neutral pH [17], [41].

**Figure 9:**
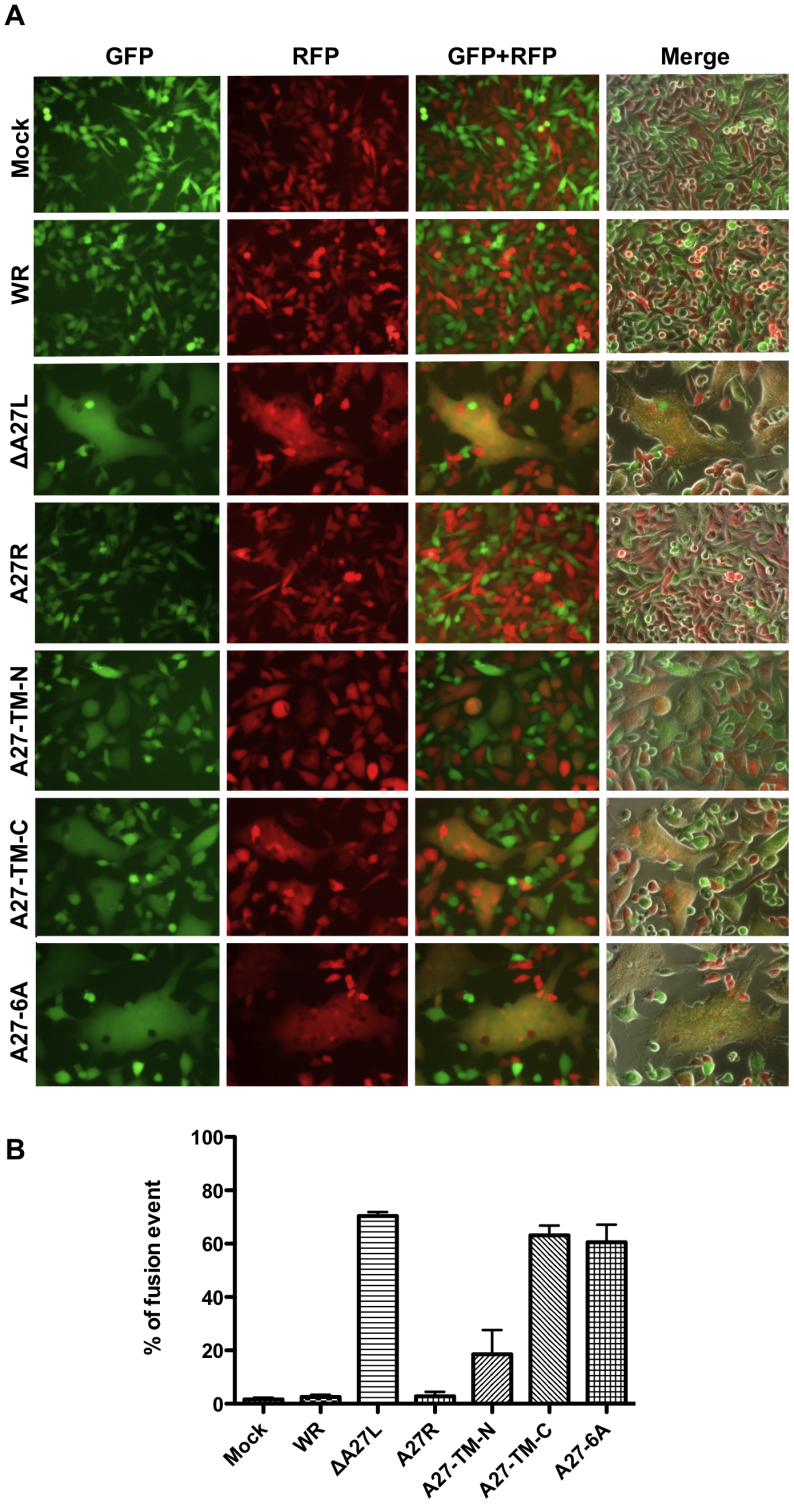
WRΔA27L, WR-A27-TM-N, WR-A27-TM-C, and WR-A27-6A induce cell-to-cell membrane fusion on L cells at neutral pH. (A) L cells expressing GFP or RFP (1∶1 mixture) were either mock-infected or infected with WR, WRΔA27L, WR-A27R, WR-A27-TM-N, WR-A27-TM-C, and WR-A27-6A viruses at an MOI of 50 PFU/cell at 37°C for 30 min and monitored for cell-to-cell fusion at a neutral pH, as described in Materials and Methods. Cell images were photographed at 2 h post-infection. (B) Quantification of cell-to-cell fusion at neutral pH. The percentage of cells containing both GFP and RFP fluorescence was quantified as cell-cell fusion using Axio Vision Rel. 4.8 with a Zeiss Axiovert fluorescence microscope.

Using purified vaccinia MV particles from WR-A27-TM-N, WR-A27-TM-C, and WR-A27-6A infections, we investigated whether these MV particles trigger plasma membrane fusion at neutral pH. L cells expressing either green fluorescent protein (GFP) or red fluorescent protein (RFP) were mixed at a 1∶1 ratio and subsequently infected with each purified MV at an MOI of 50 PFU/cell. Cell fusion was monitored at 1–2 h post-infection, as previously described [17]. WR virus infection did not trigger cell-cell fusion and GFP- and RFP-expressing cells were well separated, similar to mock-infected cells (Figure 9A). On the other hand, WRΔA27L virus proceeded readily with plasma membrane fusion, resulting in gigantic fused cells that co-expressed GFP and RFP. As expected, WR-A27R virus behaved like WR virus and did not trigger cell-cell fusion. WR-A27-TM-N infection exhibited an intermediate phenotype, with multi-nucleate fused cells of smaller sizes than those observed with WRΔA27L. Finally, WR-A27-TM-C and WR-A27-6A triggered gigantic cell-cell fusion similar to WRΔA27L. Quantitative cell-cell fusion data are presented in Figure 9B. This finding is somewhat anticipated because WR-A27-TM-C and WR-A27-6A MV particles contained neither A26 nor A27 proteins. Taken together, the above results revealed that A27 self-assembly structure is critical for A26 protein incorporation into MV particles to suppress plasma membrane fusion at neutral pH.

## Discussion

Vaccinia A27 protein plays multiple roles in the vaccinia virus life cycle, including binding to HS, membrane fusion regulation, and the mediation of MV transport to form EV. In previous studies, we characterized the secondary structure of soluble T7-tA27 using CD and NMR spectroscopy, and we were able to predict the secondary structure of the T7-tA27 monomer [29]. Nonetheless, previously the data only allowed us to propose a “molecular model” of A27 protein, which is not a real 3D NMR structure.

In contrast, the current crystal structure has revealed that tA27 protein interaction relies on hydrophobic interactions of the CCD, including L47, L51, and L54 at the NTR interface and I68, N75, and L82 at the CTR interface. Structure modeling of the CCD domain of A27 orthologs demonstrates conservation of structural folding, implying that the CCD domain evolved to form similar trimers in order to conserve functions. Although we used the terms NTR and CTR interface to differentiate these two contact sites in the crystal structure, both sites remain conserved in the full-length A27 protein.

The crystal structure is consistent with previous *in vitro* studies of recombinant T7-tA27 protein, which demonstrated that the rigid hydrophobic CCD is essential for protein assembly in solution [31]. However, given the fact that tA27 assembles into a rod-like coiled-coil trimer in the crystalline state, the conventional molecular weight estimation employed in previous studies using SEC analysis (which assumes that the target proteins adopt compact and globular folds) might be erroneous [31]. In the present study, we employ two independent biophysical methods (AUC-SV and SEC/MALS) to determine the molecular weight of proteins without assuming that the protein of interest is compact and globular [39]. Both measurements showed that tA27 is monomeric at a low protein concentration, and a dimeric population emerges when the protein concentration is increased. Thus, the discrepancy between our current data and the previously reported data [31] may be largely attributed to the non-globular structure of tA27, which contains a highly disordered N-terminal part and a highly elongated C-terminal helix that render the molecular weight estimation by conventional SEC unsuitable. It is not uncommon for the retention volume of a loosely structured protein or an intrinsically disordered protein to deviate from its expected molecular weight, leading to an over-estimation of the molecular weight in spite of the use of protein standards to calibrate the SEC analysis [42]. While the AUC-SV and SEC/MALS results may seem to contradict the crystallographic findings, it is important to note that the SEC/MALS and AUC measurements were performed in solution, while protein crystallization is by default an oligomerization process, which has a very high local concentration. As tA27 exhibits increased oligomerization propensity at high protein concentrations, it is plausible that the formation of a trimer/hexamer as seen in the crystal structure occurs *in vivo* where the local concentration is elevated. Indeed, our immunoblot analysis identified the presence of dimeric and trimeric A27 *in vivo* together with the formation of A26-A27 complexes.

The recombinant viruses provided strong evidence supporting the hypothesis that these residues residing on the NTR and CTR interfaces are involved in A27 dimer/trimer formation *in vivo*. Furthermore, the ability of A27 to self-assemble appeared to be coupled with its adaptor function for anchoring A26 proteins on MV and A26 stability. While the wild type MV contained A26-A27 protein complexes of 70-kDa and 90-kDa on MV; the TM-N mutant MV only contained the 90-kDa form, and the TM-C and 6A mutant MV particles lacked both. Consequently, these mutant MV particles, with little or no A26 fusion suppressor on the cell surface, triggered plasma membrane fusion at neutral pH. In fact, the endocytic route of the wild type WR MV into HeLa cells was sensitive to bafilomycin (an inhibitor of endosome acidification); the entry of WRΔA27L and all three A27 mutants (WR-A27-TM-N, WR-A27-TM-C, and WR-A27-6A) became resistant to bafilomycin (data not shown).

In the literature, the role of A27 protein in membrane fusion has been puzzling. Earlier studies provided evidence to support the hypothesis that A27 is the viral fusion protein [10], [11], [12], [14], [43]. Other studies argued against it [15], [25], [26], [44], particularly considering that A27 protein is inessential for vaccinia MV infectivity in contrast, a viral EFC containing 12 viral components is essential for virus-mediated membrane fusion [15], [44]. Indeed, the tA27 crystal structure differs from a typical viral fusion protein, as previously proposed [13]–;. Moreover, using these A27 contact interface mutant viruses, we confirmed that the assembly of A27 protein affects formation of the A26A27 protein complex, without which the A26 protein became destabilized the resulting MV particles inherited such changes, and in the next round the virus entry pathway is altered accordingly. Together, our results support that A27 protein is not the viral fusion protein, and instead regulates viral fusion *indirectly*– by acting as an adaptor to control A26 protein incorporation into MV particles. The current tA27 structure study reinforced our idea that A26A27 protein complex assembly acts as the molecular basis of membrane fusion regulation. Finally, since the GAG binding activity of A27 protein also depends on oligomer formation [29], future crystallization of the tA27 protein structure in complex with the ligand or other viral proteins will be of importance to help dissect the A27 functions during vaccinia virus entry.

## Materials and Methods

### Cell cultures, reagents and viruses

N-Ethylmaleimide (NEM) was purchased from Sigma, Inc. BSC40, BSC40-A27, HeLa, 293T, and L cells were cultured in Dulbecco's modified Eagle's medium (DMEM) supplemented with 10% fetal bovine serum (Invitrogen). The Western Reserve (WR) strain of vaccinia virus was used as described previously [8]. Purification of vaccinia virus MV was performed through a 36% sucrose cushion followed by CsCl gradient centrifugation as described previously [45], [46]. Anti-A26, anti-A27, anti-H3 and anti-D8 antibodies were described previously [6], [17], [23].

### Cloning and *in vitro* mutagenesis

The plasmid construct that expressed recombinant tA27 protein (aa 21–84) containing C71A/C72A has been described previously [29], [30], [31]. To obtain recombinant tA27 protein for crystallization, the A27L insert encoding A27 (aa 21–84)-C71/72A (tA27) protein was amplified by PCR and subcloned into pET-32 Xa/LIC (Novagen) to obtain pET-32-tA27. One additional His_6_ tag was subsequently inserted to increase the binding affinity with the Ni-NTA column for affinity chromatography.

To obtain recombinant tA27 protein for *in vitro* biophysical analysis, a factor Xa cutting site followed by three extra nucleotides (TGG, encoding tryptophan) was inserted immediately before the tA27 (aa 21–84)-C71A/C72A ORF and subcloned into pET-32 EK/LIC (Novagen) to obtain pET-32-tA27-WT. Plasmids containing tA27-TM-N, tA27-TM-C, and tA27-6A mutations (Table S3) were subsequently generated using two-step PCR and the sequences were confirmed by DNA sequencing.

### Protein expression and purification of recombinant tA27

pET-32-tA27 DNA construct was expressed in the *E. coli* BL21 (*DE3*) (Novagen) strain and purified based on the same procedures described below. Bacterial cultures were grown in LB medium containing 100 µg/ml carbenicillin at 30°C and induced with 0.5 mM isopropyl-β-thiogalactopyranoside (IPTG) at 15°C overnight until the *A*_600_ reached 0.8. Bacteria pellets were harvested by centrifugation at 6,000 *g* for 15 min at 4°C and resuspended in extraction buffer [50 mM Tris, pH 8.0, 20 mM imidazole, 0.15 M NaCl, 10% (w/v) glycerol, 10 mM MgCl_2_, 2 ng/ml of benzonase (Novagen), and EDTA-free protease inhibitor cocktail (Roche)]. The pellets were lysed in Cell Disruption Solutions (Constant Systems) and centrifuged at 30,000 *g* for 45 min at 4°C to remove insoluble debris. Proteins were purified using Ni-NTA affinity chromatography (GE Healthcare) and eluted with a linear gradient to 100% (v/v) elution buffer 25 mM Tris, pH 7.5, [0.25 M imidazole, 0.15 M NaCl, 10 (w/v) glycerol[. The eluted tA27 sample was dialyzed twice against 5 liters of buffer 25 mM Tris, pH 7.5 and 0.15 M NaCl and then subjected to Factor Xa digestion to remove the histidine-tagged fusion protein. The untagged tA27 was purified using another Ni-NTA affinity chromatography column (GE Healthcare) followed by gel filtration (HiLoad 16/600 Superdex 75, GE Healthcare) in 25 mM Tris, pH 7.5 and 0.15 M NaCl. SDS-PAGE confirmed the purity of tA27 above 99% (Figure S1). Recombinant tA27-WT and mutant proteins including tA27-TM-N, tA27-TM-C, and tA27-6A were purified using the same procedures.

### Crystallization and data collection

tA27 was crystallized in hanging drops using the vapor diffusion method at 20°C for 1 to 2 weeks by mixing 2 µl of protein solution (5 mg/ml) with 2 µl of reservoir solution [0.1 M phosphate-citrate, pH 4.5, 0.2 M NaCl, and 45–50% (w/v) polyethylene glycol 200 and equilibrating with 0.5 ml reservoir solution. The tantalum bromide derivative crystals were obtained by soaking native tA27 crystals for 6 h in the mother liquor supplemented with 1 mM tantalum bromide cluster ]Ta_6_Br_12_]^+2^[. Crystals were directly mounted from mother liquor and immediately flash-cooled to 100 K in a stream of cold nitrogen. Diffraction data for native tA27 were collected at Taiwan Contract BL12B2 station at Spring-8 (Hyogo, Japan), and the tantalum bromide cluster derivative data were collected at BL13B1 of the National Synchrotron Radiation Research Center (Hsinchu, Taiwan) using the two-wavelength multiple-wavelength anomalous diffraction method at the tantalum edge. Diffraction data were processed and scaled using the HKL2000 package [47]. Five percent of the randomly selected diffraction data were used to calculate *R*_free_ [48]. Statistics are shown in Table 1.

### Structure determination, refinement, and validation

The SHELXD program [49] identified one cluster site per one tA27 trimer in an asymmetric unit using a two-wavelength MAD experiment (Figure S2). The cluster locations and phases were refined and calculated using SHARP [50]. Density modification and phase extension performed with DM [51] yielded an initial electron density map for automatic model building in Buccaneer [52]Å. This preliminary model served as a search template for using the molecular replacement method to determine the higher resolution structure of native tA27 at 2.2 with PHASER [53]. Manual checking and building were performed in Coot [54], and refinement was done using REFMAC [55], with non-crystallographic symmetry restraints fixed for chains A and B and translation libration screw refinement. Structure analysis and stereochemical quality were performed with MolProbity [56]. The crystallographic statistics are listed in Table 1. The superimpositions of the individual chains were performed using Least Square Fit in Coot [54];. High-quality images of the molecular structures were created with PyMOL (DeLano, 2002 http://www.pymol.org/).

### Size exclusion chromatography

The oligomerization of wild type and mutant tA27 proteins in solution was analyzed on a size exclusion column (Superdex75 10/300 GL High Performance, GE Healthcare) in 25 mM Tris, pH 7.5 and 0.15 M NaCl or pH 3.0 at a flow rate of 0.2 ml/min. The injection volume was 100 µl, containing 1 mg/ml or 9.5 mg/ml protein solution. The molecular weight of wild type and mutant tA27 proteins at pH 7.5 were calculated by comparing with those of protein molecular mass standards, including aprotinin (6.5 kDa), ribonuclease A (13.7 kDa), carbonic anhydrase (29 kDa), ovalbumin (43 kDa), conalbumin (75 kDa), and ferritin (440 kDa).

### Analytical ultracentrifugation (AUC)

Sedimentation velocity was performed using an XL-A analytical ultracentrifuge (Beckman Coulter, Fullerton, CA) with absorption optics, using an AnTi60 rotor with cells containing quartz windows. Samples and control reference buffer (approximately 400 µL) were added to double-sector centerpieces radial absorbance data at 280 nm was acquired at 3-min intervals and a rotor speed of 60,000 rpm at 20°C. Wild type and mutant tA27 protein samples (120 µM) were in buffer containing 25 mM Tris, pH 7.5, 0.15 M NaCl or pH 3.0. The buffer density and viscosity were calculated using SEDNTERP [57], and the data were analyzed using SEDFIT software [58].

### Size exclusion chromatography/multi-angle light scattering (SEC/MALS)

Absolute molecular weights were determined with static light scattering using a Wyatt Dawn Heleos II multi-angle light scattering (MALS) detector (Wyatt Technology) coupled to an AKTA Purifier UPC10 FPLC protein purification system with a Superdex 75 10/300 GL High Performance size exclusion column (GE Healthcare). tA27 protein (at concentrations of 1 mg/ml and 9.5 mg/ml) was applied to the size exclusion column in a buffer containing 25 mM Tris, pH 7.5, 0.15 M NaCl, and 0.02% NaN_3_, at a flow rate of 0.5 ml/min. A BSA sample (3.2 mg/ml) was used as a reference to calibrate the system. The absolute molecular weights of individual peaks in the size exclusion chromatogram were determined using the static light scattering (SLS) data in conjunction with the refractive index measurements (Wyatt Optilab rEX, connected downstream of the LS detector). A standard value of refractive index (d*n*/d* = c*η = °0.185 ml/g) was used for proteins, and the buffer viscosity 1.0226 cP at 25C was calculated using SEDNTERP. The value of the reference refractive index, 1.3452 RIU, was taken directly from the measurement of the Wyatt Optilab rEX with buffer alone passing through the reference cell.

### Generation of a stable BSC40 cell line expressing A27 protein

To express vaccinia WR full-length A27 protein in BSC40 cells, the WR A27L ORF was codon-optimized for eukaryotic expression (Gene Script, Inc.) and cloned into the plasmid pLKO-AS3.1-EGFP3′ (National RNAi Core, Academia Sinica, Taiwan). 293T cells were transfected with pLKO-AS3.1-EGFP3′-A27, pCMV-ΔR8.91, and pMD.G (National RNAi Core, Academia Sinica, Taiwan), and the supernatant was collected at 48 h post-infection and subsequently applied to BSC40 cells for lentiviral transduction. These transduced cells expressing EGFP were collected using a FACS Vantage SE/DiVa. The sorted EGFP-positive cells were grown and re-sorted to enrich the population of high-EGFP-expressing cells. The process was repeated three times, and the cells were used in plaque assays.

### Construction of WRΔA27L, WR-A27L-Reventant (WR-A27R), WR-A27-TM-N, WR-A27-TM-C, and WR-A27-6A recombinant viruses

To construct a plasmid for generating the WRΔA27L virus, A26L and A28-A29L ORF sequences were amplified out of viral genomic DNA using PCR and cloned into pBluescript vector as flanking sequences. A *luc-gpt* cassette was subsequently inserted between the A26L and A28-A29L sequences, resulting in a plasmid pA26L/*luc-gpt*/A28L-A29L-KS (−). The plasmid was transfected into 293T cells that were subsequently infected with vaccinia virus (WR), and the lysates were harvested at 3 days post infection for isolation of WRΔA27L virus in 1% agar containing 25 µg/ml mycophenolic acid, 250 µg/ml xanthine, and 15 µg/ml hypoxanthine as described previously [59].

*In vitro* mutagenesis was performed using the QuikChange site-directed mutagenesis kit (Stratagene) to obtain mutant A27L ORFs encoding the full-length A27-TM-N, A27-TM-C and A27-6A. The wild type A27L and the mutant A27-TM-N, A27-TM-C, and A27-6A ORFs were cloned into pMJ601 to produce pMJ601-A27R, pMJ601-A27-TM-N, WR-A27-TM-C, and WR-A27L-6A, respectively, all of which were confirmed by DNA sequencing. The resulting plasmids were transfected individually transfected into 293T cells that were subsequently infected with WRΔA27L, and cultured for 2 to 3 days, and harvested for recombinant virus isolation by with three rounds of plaque purification in 1% agar with 150 µg/ml of X-Gal (5-bromo-4-chloro-3-indolyl-β-D-galactopyranoside).

### Cell fusion assay (fusion from without)

Cell fusion assays were performed as previously described [17]. In brief, L cells expressing GFP or RFP were mixed at a 1∶1 ratio and seeded in 96-well plates (4×10^4^/well). The next day, cells were pretreated with 40 µg/ml cordycepin (Sigma) for 60 min and subsequently infected with CsCl-purified wild-type (wt) WR, WRΔA27L, WR-A27R, WR-A27-TM-N, WR-A27-TM-C, and WR-A27-6A viruses at an MOI of 50 PFU/cell in duplicate. After infection at 37°C for 30 min, cells were incubated at 37°C in the presence of (40 µg/ml) cordycepin and photographed at 2 h post-infection using a Zeiss Axiovert fluorescence microscope. The percentage of cells containing both GFP and RFP fluorescence was quantified as cell-cell fusion using Axio Vision Rel. 4.8 software.

### Plaque formation on BSC40 and BSC40-A27 cells

Freshly confluent cells were infected with WR, WRAΔ27L, WR-A27R, WR-A27-TM-N, WR-A27-TM-C, and WR-A27-6A viruses at 37°C for 1 h and subsequently cultured in growth medium containing 1% agar. At 2 days post-infection, the cells were fixed and stained with either 300 µg/ml X-gal or 1% crystal violet/20% EtOH (Sigma).

### Immunoblot analysis

To avoid artificial reduction and disulfide bond rearrangement during cell rupture, cell lysates were prepared in the presence of an alkylating agent, NEM, using methods described previously [23]. Briefly, cells were infected with various viruses at an MOI of 5 PFU/cell for 1 h at 37°C and harvested at 24 h post-infection. For non-reducing gel analysis, cells were placed on ice and washed twice with freshly prepared cold phosphate-buffered saline (PBS) containing 20 mM NEM (Sigma) and subsequently mixed with an equal volume of SDS-polyacrylamide gel electrophoresis (PAGE) loading buffer (150 mM Tris-HCl, pH 6.8; 20% glycerol; 4% SDS; 0.04% bromophenol blue), boiled for 10 min, and loaded on NuPAGE 4% to 12% Bis-Tris denaturing gels (Invitrogen). For reducing gel analysis, SDS-PAGE loading buffer was supplemented with 5% 2-mercaptoethanol and cell lysates were separated on 10% and 15% SDS-PAGE gels. Proteins were subsequently transferred onto nitrocellulose membranes for immunoblot analysis with anti-A26 (1∶1,000), anti-A27 (1∶5,000), anti-H3 (1∶1,000) and anti-D8 (1∶5,000) antibodies.

### Accession number

Coordinates and structural factors of tA27 have been deposited in the Protein Data Bank with 3VOP accession number.

## Supporting Information

Figure S1 **Analysis of purified tA27 on the reduced SDS-PAGE.** Lane 1: molecular mass standard; 2: purified tA27.
(TIF)

Figure S2 **Experimental phasing.** Anomalous difference Fourier map for [Ta_6_Br_12_]^+2^ cluster sites, shown as green meshes, are calculated from tantalum bromide cluster derivative X-ray diffraction data.
(TIF)

Figure S3 **Electron density maps for tA27 structure.** The ribbon diagram of tA27 are colored in blue (chain A), cyan (chain B), and magenta (chain C). The 2|*F*_O_|-|*F*_C_| electron density maps were contoured at 1.0 σ level as green meshes.
(TIF)

Figure S4 **Superimposition of three individual chains of tA27.** Structural comparisons of chain A (blue), chain B (cyan), and chain C (Magenta) are shown as ribbon diagram. The N-terminus of helices point toward the top and the C-terminus of those point toward the bottom.
(TIF)

Table S1 **Class I virus fusion proteins.**
(DOCX)

Table S2 **Analyses of stable tA27 protein assembly in solution.**
(DOCX)

Table S3 **Size exclusion chromatography (SEC) of wild type and mutant tA27 proteins.**
(DOCX)

## References

[1] Goebel SJ, Johnson GP, Perkus ME, Davis SW, Winslow JP, et al (1990) The complete DNA sequence of vaccinia virus. Virology 179: 247––266, 517263. 2219722 10.1016/0042-6822(90)90294-2

[2] Condit RC, Moussatche N, Traktman P (2006) In a nutshell: structure and assembly of the vaccinia virion. Adv Virus Res 66: 31–124. 16877059 10.1016/S0065-3527(06)66002-8

[3] Chung CS, Chen CH, Ho MY, Huang CY, Liao CL, et al (2006) Vaccinia virus proteome: identification of proteins in vaccinia virus intracellular mature virion particles. J Virol 80: 2127–2140. 16474121 10.1128/JVI.80.5.2127-2140.2006 PMC1395410

[4] Resch W, Hixson KK, Moore RJ, Lipton MS, Moss B (2007) Protein composition of the vaccinia virus mature virion. Virology 358: 233–247. 17005230 10.1016/j.virol.2006.08.025

[5] Chung CS, Hsiao JC, Chang YS, Chang W (1998) A27L protein mediates vaccinia virus interaction with cell surface heparan sulfate. J Virol 72: 1577–1585. 9445060 10.1128/jvi.72.2.1577-1585.1998 PMC124638

[6] Hsiao JC, Chung CS, Chang W (1999) Vaccinia virus envelope D8L protein binds to cell surface chondroitin sulfate and mediates the adsorption of intracellular mature virions to cells. J Virol 73: 8750–8761. 10482629 10.1128/jvi.73.10.8750-8761.1999 PMC112896

[7] Lin CL, Chung CS, Heine HG, Chang W (2000) Vaccinia virus envelope H3L protein binds to cell surface heparan sulfate and is important for intracellular mature virion morphogenesis and virus infection in vitro and in vivo. J Virol 74: 3353–3365. 10708453 10.1128/jvi.74.7.3353-3365.2000 PMC111837

[8] Chiu WL, Lin CL, Yang MH, Tzou DL, Chang W (2007) Vaccinia virus 4c (A26L) protein on intracellular mature virus binds to the extracellular cellular matrix laminin. J Virol 81: 2149–2157. 17166913 10.1128/JVI.02302-06 PMC1865921

[9] Rodriguez JF, Janeczko R, Esteban M (1985) Isolation and characterization of neutralizing monoclonal antibodies to vaccinia virus. J Virol 56: 482–488. 4057358 10.1128/jvi.56.2.482-488.1985 PMC252603

[10] Rodriguez JF, Esteban M (1987) Mapping and nucleotide sequence of the vaccinia virus gene that encodes a 14-kilodalton fusion protein. J Virol 61: 3550–3554. 2822962 10.1128/jvi.61.11.3550-3554.1987 PMC255954

[11] Rodriguez JF, Paez E, Esteban M (1987) A 14,000-Mr envelope protein of vaccinia virus is involved in cell fusion and forms covalently linked trimers. J Virol 61: 395–404. 3806791 10.1128/jvi.61.2.395-404.1987 PMC253962

[12] Gong SC, Lai CF, Esteban M (1990) Vaccinia virus induces cell fusion at acid pH and this activity is mediated by the N-terminus of the 14-kDa virus envelope protein. Virology 178: 81–91. 2389560 10.1016/0042-6822(90)90381-z

[13] Vazquez MI, Rivas G, Cregut D, Serrano L, Esteban M (1998) The vaccinia virus 14-kilodalton (A27L) fusion protein forms a triple coiled-coil structure and interacts with the 21-kilodalton (A17L) virus membrane protein through a C-terminal alpha-helix. J Virol 72: 10126–10137. 9811753 10.1128/jvi.72.12.10126-10137.1998 PMC110549

[14] Kochan G, Escors D, Gonzalez JM, Casasnovas JM, Esteban M (2008) –Membrane cell fusion activity of the vaccinia virus A17A27 protein complex. Cell Microbiol 10: 149–164. 17708756 10.1111/j.1462-5822.2007.01026.x

[15] Moss B (2012) ?Poxvirus cell entry: how many proteins does it take Viruses 4: 688–707. 22754644 10.3390/v4050688 PMC3386626

[16] Whitbeck JC, Foo CH, Ponce de Leon M, Eisenberg RJ, Cohen GH (2009) Vaccinia virus exhibits cell-type-dependent entry characteristics. Virology 385: 383–391. 19162290 10.1016/j.virol.2008.12.029 PMC4041486

[17] Chang SJ, Chang YX, Izmailyan R, Tang YL, Chang W (2010) Vaccinia virus A25 and A26 proteins are fusion suppressors for mature virions and determine strain-specific virus entry pathways into HeLa, CHO-K1, and L cells. Journal of virology 84: 8422–8432. 20538855 10.1128/JVI.00599-10 PMC2919003

[18] Bengali Z, Satheshkumar PS, Moss B (2012) Orthopoxvirus species and strain differences in cell entry. Virology 433: 506–512. 22999097 10.1016/j.virol.2012.08.044 PMC3470877

[19] Izmailyan R, Hsao JC, Chung CS, Chen CH, Hsu PW, et al (2012) Integrin beta1 mediates vaccinia virus entry through activation of PI3K/Akt signaling. J Virol 86: 6677–6687. 22496232 10.1128/JVI.06860-11 PMC3393588

[20] Schroeder N, Chung CS, Chen CH, Liao CL, Chang W (2012) The lipid raft-associated protein CD98 is required for vaccinia virus endocytosis. J Virol 86: 4868–4882. 22345471 10.1128/JVI.06610-11 PMC3347329

[21] Chang SJ, Shih AC, Tang YL, Chang W (2012) Vaccinia mature virus fusion regulator A26 protein binds to A16 and G9 proteins of the viral entry fusion complex and dissociates from mature virions at low pH. J Virol 86: 3809–18. 22278246 10.1128/JVI.06081-11 PMC3302531

[22] Howard AR, Senkevich TG, Moss B (2008) Vaccinia virus A26 and A27 proteins form a stable complex tethered to mature virions by association with the A17 transmembrane protein. J Virol 82: 12384–12391. 18842719 10.1128/JVI.01524-08 PMC2593336

[23] Ching YC, Chung CS, Huang CY, Hsia Y, Tang YL, et al (2009) Disulfide bond formation at the C termini of vaccinia virus A26 and A27 proteins does not require viral redox enzymes and suppresses glycosaminoglycan-mediated cell fusion. J Virol 83: 6464–6476. 19369327 10.1128/JVI.02295-08 PMC2698572

[24] Rodriguez D, Rodriguez JR, Esteban M (1993) The vaccinia virus 14-kilodalton fusion protein forms a stable complex with the processed protein encoded by the vaccinia virus A17L gene. J Virol 67: 3435–3440. 8497059 10.1128/jvi.67.6.3435-3440.1993 PMC237688

[25] Sanderson CM, Hollinshead M, Smith GL (2000) The vaccinia virus A27L protein is needed for the microtubule-dependent transport of intracellular mature virus particles. J Gen Virol 81: 47–58. 10640541 10.1099/0022-1317-81-1-47

[26] Ward BM (2005) Visualization and characterization of the intracellular movement of vaccinia virus intracellular mature virions. J Virol 79: 4755–4763. 15795261 10.1128/JVI.79.8.4755-4763.2005 PMC1069544

[27] Rodriguez JF, Smith GL (1990) Inducible gene expression from vaccinia virus vectors. Virology 177: 239–250. 2191497 10.1016/0042-6822(90)90477-9

[28] Rodriguez JF, Smith GL (1990) IPTG-dependent vaccinia virus: identification of a virus protein enabling virion envelopment by Golgi membrane and egress. Nucleic Acids Res 18: 5347–5351. 2216706 10.1093/nar/18.18.5347 PMC332208

[29] Lin TH, Chia CM, Hsiao JC, Chang W, Ku CC, et al (2002) Structural analysis of the extracellular domain of vaccinia virus envelope protein, A27L, by NMR and CD spectroscopy. J Biol Chem 277: 20949–20959. 11901147 10.1074/jbc.M110403200

[30] Shih PC, Yang MS, Lin SC, Ho Y, Hsiao JC, et al (2009) A turn-like structure “KKPE” segment mediates the specific binding of viral protein A27 to heparin and heparan sulfate on cell surfaces. J Biol Chem 284: 36535–36546. 19858217 10.1074/jbc.M109.037267 PMC2794769

[31] Ho Y, Hsiao JC, Yang MH, Chung CS, Peng YC, et al (2005) The oligomeric structure of vaccinia viral envelope protein A27L is essential for binding to heparin and heparan sulfates on cell surfaces: a structural and functional approach using site-specific mutagenesis. J Mol Biol 349: 1060–1071. 15913650 10.1016/j.jmb.2005.04.024

[32] Hsiao JC, Chung CS, Chang W (1998) Cell surface proteoglycans are necessary for A27L protein-mediated cell fusion: identification of the N-terminal region of A27L protein as the glycosaminoglycan-binding domain. J Virol 72: 8374–8379. 9733888 10.1128/jvi.72.10.8374-8379.1998 PMC110218

[33] Krissinel E (2010) 'Crystal contacts as natures docking solutions. J Comput Chem 31: 133–143. 19421996 10.1002/jcc.21303

[34] Fry EE, Lea SM, Jackson T, Newman JW, Ellard FM, et al (1999) The structure and function of a foot-and-mouth disease virus-oligosaccharide receptor complex. Embo J 18: 543–554. 9927414 10.1093/emboj/18.3.543 PMC1171147

[35] Xie Q, Bu W, Bhatia S, Hare J, Somasundaram T, et al (2002) The atomic structure of adeno-associated virus (AAV-2), a vector for human gene therapy. Proc Natl Acad Sci U S A 99: 10405–10410. 12136130 10.1073/pnas.162250899 PMC124927

[36] Opie SR, Warrington KH Jr, Agbandje-McKenna M, Zolotukhin S, Muzyczka N (2003) Identification of amino acid residues in the capsid proteins of adeno-associated virus type 2 that contribute to heparan sulfate proteoglycan binding. J Virol 77: 6995–7006. 12768018 10.1128/JVI.77.12.6995-7006.2003 PMC156206

[37] Kern A, Schmidt K, Leder C, Muller OJ, Wobus CE, et al (2003) Identification of a heparin-binding motif on adeno-associated virus type 2 capsids. J Virol 77: 11072–11081. 14512555 10.1128/JVI.77.20.11072-11081.2003 PMC224995

[38] Vazquez MI, Esteban M (1999) Identification of functional domains in the 14-kilodalton envelope protein (A27L) of vaccinia virus. J Virol 73: 9098–9109. 10516016 10.1128/jvi.73.11.9098-9109.1999 PMC112942

[39] van Holde KE, Johnson WC, Ho PS (2005) Principles of Physical Biochemistry Upper Saddle River: Prentice-Hall, Inc.

[40] Davison AJ, Moss B (1990) New vaccinia virus recombination plasmids incorporating a synthetic late promoter for high level expression of foreign proteins. Nucleic Acids Res 18: 4285–4286. 2377486 10.1093/nar/18.14.4285 PMC331224

[41] Chang SJ, Shih AC, Tang YL, Chang W (2012) Vaccinia mature virus fusion regulator A26 protein binds to A16 and G9 proteins of the viral entry fusion complex and dissociates from mature virions at low pH. J Virol 86: 3809–3818. 22278246 10.1128/JVI.06081-11 PMC3302531

[42] Fauvet B, Mbefo MK, Fares MB, Desobry C, Michael S, et al (2012) alpha-Synuclein in central nervous system and from erythrocytes, mammalian cells, and Escherichia coli exists predominantly as disordered monomer. J Biol Chem 287: 15345–15364. 22315227 10.1074/jbc.M111.318949 PMC3346117

[43] Vazquez M-I, Rivas G, Cregut D, Serrano L, Esteban M (1998) αThe vaccinia virus 14-Kilodalton (A27L) fusion protein forms a triple coiled-coil structure and interacts with the 21-Kilodalton (A17L) virus membrane protein through a C-terminal -helix. J Virol 72: 10126–10137. 9811753 10.1128/jvi.72.12.10126-10137.1998 PMC110549

[44] Senkevich TG, Ojeda S, Townsley A, Nelson GE, Moss B (2005) Poxvirus multiprotein entry-fusion complex. Proc Natl Acad Sci U S A 102: 18572–18577. 16339313 10.1073/pnas.0509239102 PMC1309049

[45] Engelstad M, Smith GL (1993) The vaccinia virus 42-kDa envelope protein is required for the envelopment and egress of extracellular virus and for virus virulence. Virology 194: 627–637. 8503178 10.1006/viro.1993.1302

[46] Payne L (1978) Polypeptide composition of extracellular enveloped vaccinia virus. J Virol 27: 28–37. 691112 10.1128/jvi.27.1.28-37.1978 PMC354137

[47] –Otwinowski Z, Minor W (1997) Processing of X-ray diffraction data collected in oscillation mode. Macromolecular Crystallography, Pt A. San Diego: Academic Press Inc. pp. 307326. 10.1016/S0076-6879(97)76066-X 27754618

[48] Brunger AT (1993) Assessment of phase accuracy by cross validation: the free R value. Methods and applications. Acta Crystallogr D Biol Crystallogr 49: 24–36. 15299543 10.1107/S0907444992007352

[49] Sheldrick GM (2010) Experimental phasing with SHELXC/D/E: combining chain tracing with density modification. Acta Crystallogr D Biol Crystallogr 66: 479–485. 20383001 10.1107/S0907444909038360 PMC2852312

[50] Vonrhein C, Blanc E, Roversi P, Bricogne G (2007) Automated structure solution with autoSHARP. Methods Mol Biol 364: 215–230. 17172768 10.1385/1-59745-266-1:215

[51] Cowtan K (1999) Error estimation and bias correction in phase-improvement calculations. Acta Crystallogr D Biol Crystallogr 55: 1555–1567. 10489450 10.1107/s0907444999007416

[52] Cowtan K (2006) The Buccaneer software for automated model building. 1. Tracing protein chains. Acta Crystallogr D Biol Crystallogr 62: 1002–1011. 16929101 10.1107/S0907444906022116

[53] McCoy AJ (2007) Solving structures of protein complexes by molecular replacement with Phaser. Acta Crystallogr D Biol Crystallogr 63: 32–41. 17164524 10.1107/S0907444906045975 PMC2483468

[54] Emsley P, Cowtan K (2004) Coot: model-building tools for molecular graphics. Acta Crystallogr D Biol Crystallogr 60: 2126–2132. 15572765 10.1107/S0907444904019158

[55] Murshudov GN, Vagin AA, Dodson EJ (1997) Refinement of macromolecular structures by the maximum-likelihood method. Acta Crystallogr D Biol Crystallogr 53: 240–255. 15299926 10.1107/S0907444996012255

[56] Chen VB, Arendall WB (2010) MolProbity: all-atom structure validation for macromolecular crystallography. Acta Crystallogr D Biol Crystallogr 66: 12–21. 20057044 10.1107/S0907444909042073 PMC2803126

[57] Hayes D, Laue T, Philo J (1995) Program Sednterp: sedimentation interpretation program. Durham, NH: University of New Hampshire.

[58] Schuck P (2000) Size-distribution analysis of macromolecules by sedimentation velocity ultracentrifugation and lamm equation modeling. Biophys J 78: 1606–1619. 10692345 10.1016/S0006-3495(00)76713-0 PMC1300758

[59] Izmailyan RA, Huang CY, Mohammad S, Isaacs SN, Chang W (2006) The envelope G3L protein is essential for entry of vaccinia virus into host cells. J Virol 80: 8402–8410. 16912291 10.1128/JVI.00624-06 PMC1563860

[60] Bullough PA, Hughson FM, Skehel JJ, Wiley DC (1994) Structure of influenza haemagglutinin at the pH of membrane fusion. Nature 371: 37–43. 8072525 10.1038/371037a0

[61] Kelley LA, Sternberg MJ (2009) Protein structure prediction on the Web: a case study using the Phyre server. Nat Protoc 4: 363–371. 19247286 10.1038/nprot.2009.2

