# Supplementary figures and images for: Crystal Structure of Vaccinia Viral A27 Protein Reveals a Novel Structure Critical for Its Function and Complex Formation with A26 Protein
Source: PLoS Pathog. 2013 Aug 22;9(8):e1003563. doi: 10.1371/journal.ppat.1003563 (PMC3749956; doi:10.1371/journal.ppat.1003563)

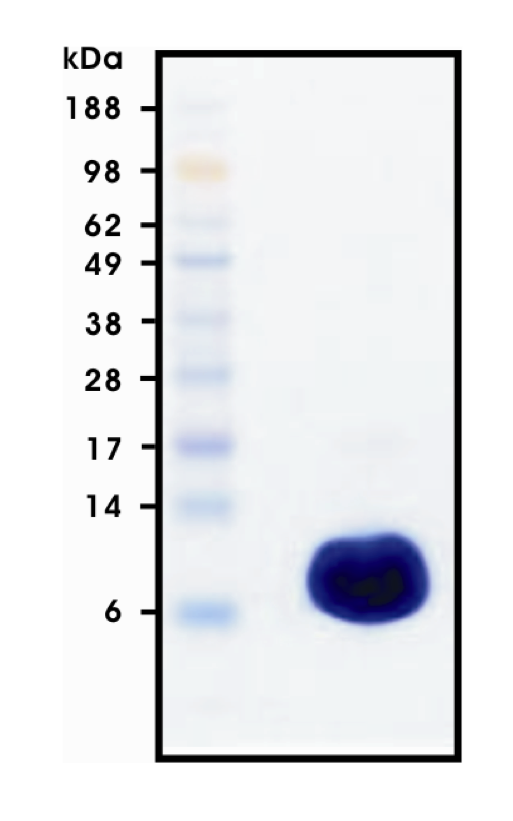

Supplement: Figure S1 — Analysis of purified tA27 on the reduced SDS-PAGE. Lane 1: molecular mass standard; 2: purified tA27. (TIF) [file ppat.1003563.s001.tif]

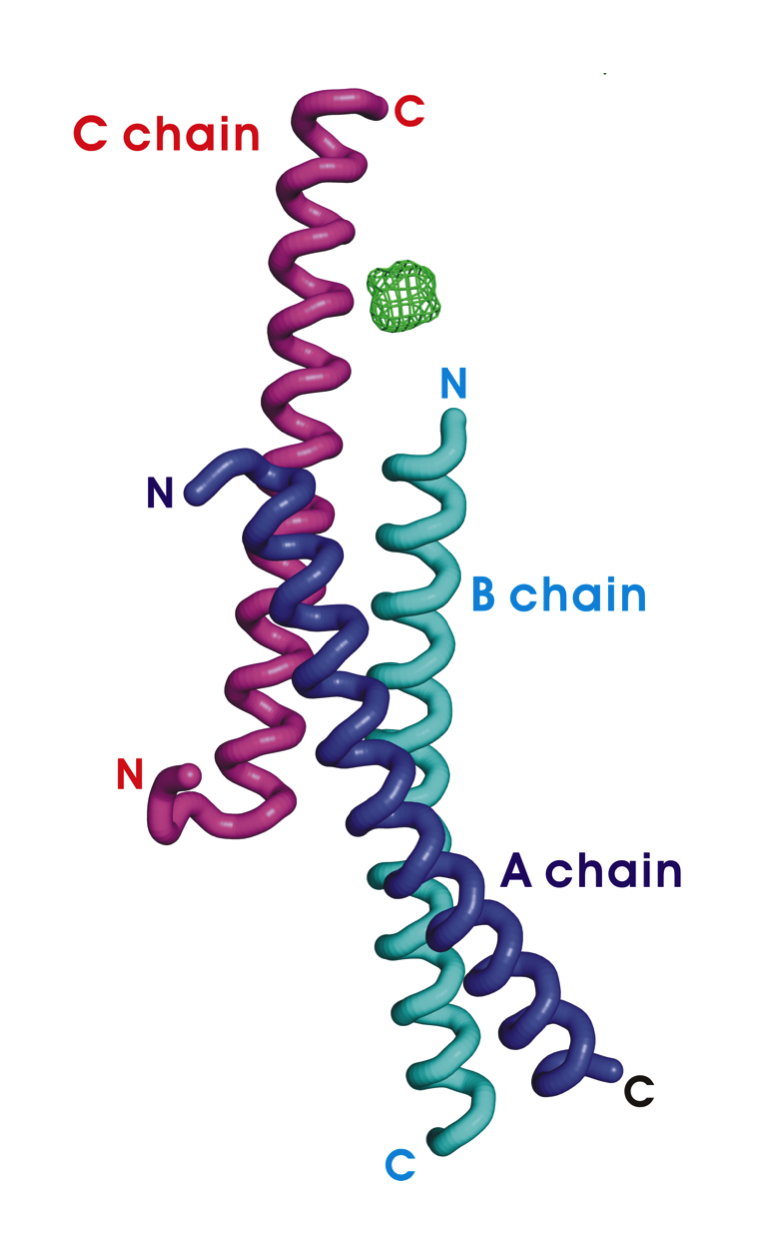

Supplement: Figure S2 — Experimental phasing. Anomalous difference Fourier map for [Ta6Br12]+2 cluster sites, shown as green meshes, are calculated from tantalum bromide cluster derivative X-ray diffraction data. (TIF) [file ppat.1003563.s002.tif]

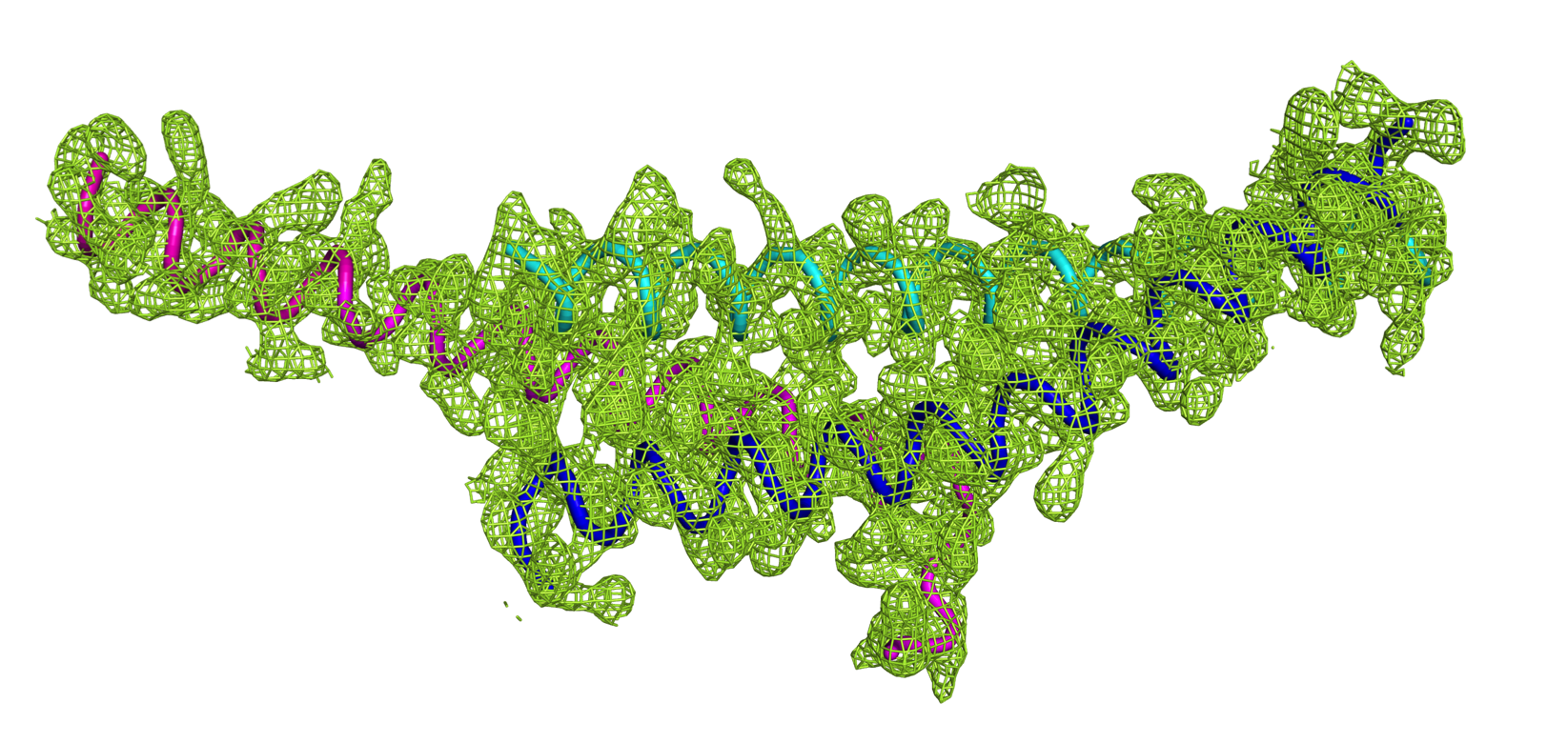

Supplement: Figure S3 — Electron density maps for tA27 structure. The ribbon diagram of tA27 are colored in blue (chain A), cyan (chain B), and magenta (chain C). The 2|F O|-|F C| electron density maps were contoured at 1.0 σ level as green meshes. (TIF) [file ppat.1003563.s003.tif]

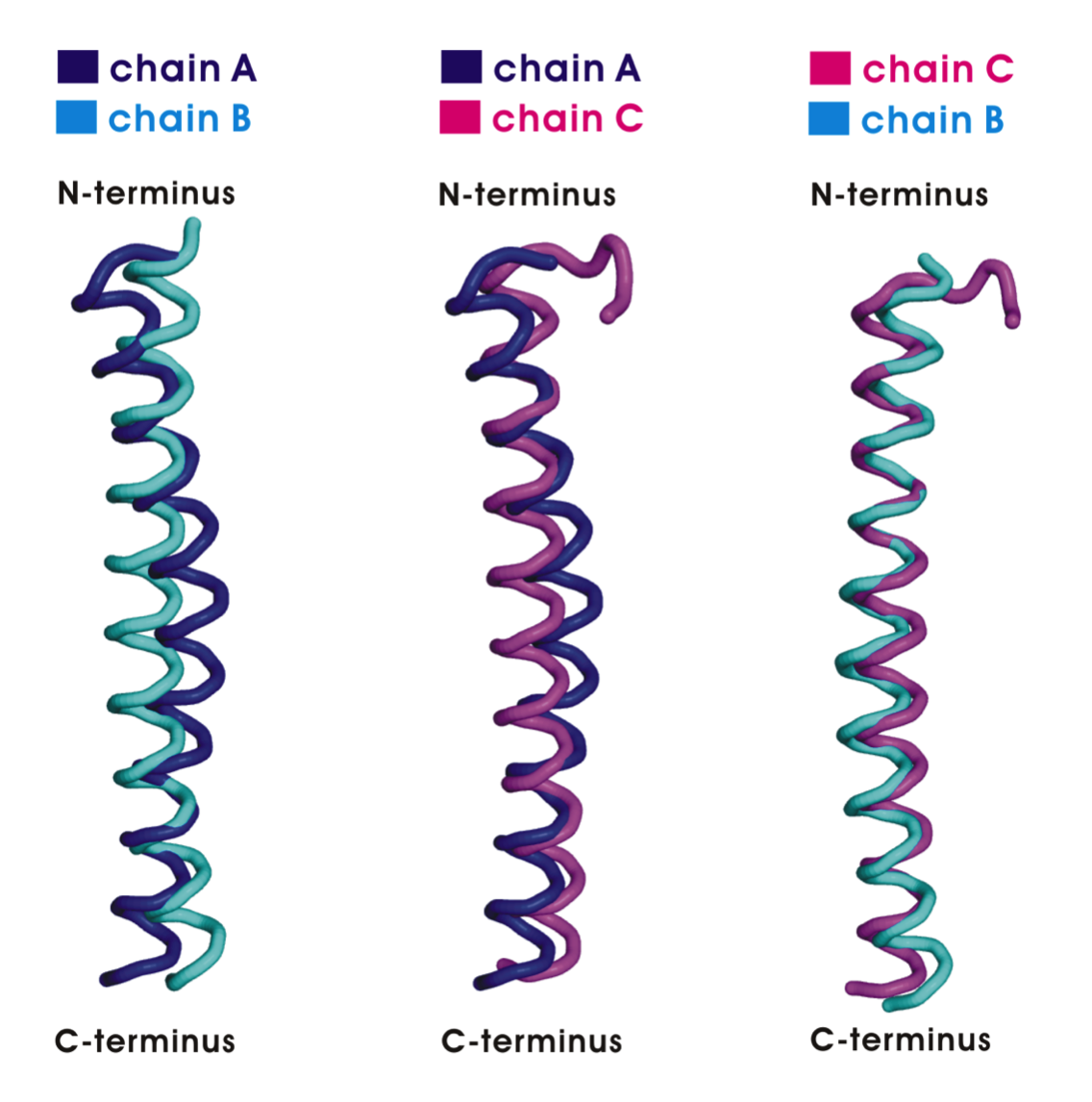

Supplement: Figure S4 — Superimposition of three individual chains of tA27. Structural comparisons of chain A (blue), chain B (cyan), and chain C (Magenta) are shown as ribbon diagram. The N-terminus of helices point toward the top and the C-terminus of those point toward the bottom. (TIF) [file ppat.1003563.s004.tif]
