# Supplementary material for: Crystal Structure of Vaccinia Viral A27 Protein Reveals a Novel Structure Critical for Its Function and Complex Formation with A26 Protein
Source: PLoS Pathog. 2013 Aug 22;9(8):e1003563. doi: 10.1371/journal.ppat.1003563 (PMC3749956; doi:10.1371/journal.ppat.1003563)
Supplement: Table S1 — Class I virus fusion proteins. (DOCX) [file ppat.1003563.s005.docx]

| **Virus family** | **Protein** | **PDB code** | **Ref** |
| --- | --- | --- | --- |
| *Orthomyxoviridae* | Influenza virus HA2 | 1HTM | [1] |
| *Retroviridae* | Human immunodeficiency virus gp41 | 1AIK | [2] |
| *Filoviridae* | Ebola virus gp2 | 2EBO | [3] |
| *Paramyxoviridae* | Simian virus 5 F | 1SVF | [4] |
| *Coronaviridae* | Severe acute respiratory syndrome-associated coronavirus S2 | 2IEQ | [5] |

Reference:

1. Bullough PA, Hughson FM, Skehel JJ, Wiley DC (1994) Structure of influenza haemagglutinin at the pH of membrane fusion. Nature 371: 37-43.

2. Chan DC, Fass D, Berger JM, Kim PS (1997) Core structure of gp41 from the HIV envelope glycoprotein. Cell 89: 263-273.

3. Malashkevich VN, Schneider BJ, McNally ML, Milhollen MA, Pang JX, et al. (1999) Core structure of the envelope glycoprotein GP2 from Ebola virus at 1.9-A resolution. Proc Natl Acad Sci U S A 96: 2662-2667.

4. Baker KA, Dutch RE, Lamb RA, Jardetzky TS (1999) Structural basis for paramyxovirus-mediated membrane fusion. Mol Cell 3: 309-319.

5. Zheng Q, Deng Y, Liu J, van der Hoek L, Berkhout B, et al. (2006) Core structure of S2 from the human coronavirus NL63 spike glycoprotein. Biochemistry 45: 15205-15215.
