# Supplementary material for: Crystal Structure of Vaccinia Viral A27 Protein Reveals a Novel Structure Critical for Its Function and Complex Formation with A26 Protein
Source: PLoS Pathog. 2013 Aug 22;9(8):e1003563. doi: 10.1371/journal.ppat.1003563 (PMC3749956; doi:10.1371/journal.ppat.1003563)
Supplement: Table S2 — Analyses of stable tA27 protein assembly in solution. (DOCX) [file ppat.1003563.s006.docx]

| **Interface** | **Buried area (Å^2^)*^a, b^*** | **Δ*G*^int^ ( kcal/mol)*^a, c^*** | **Δ*G*^diss^, (kcal/mol)*^a, d^*** |
| --- | --- | --- | --- |
| NTR | 3110 | -32.1 | 9.3 |
| CTR | 2950 | -23.8 | 2.0 |

*^a^* The value were calculated using the program Protein Interfaces, Surface, and Assemblies (PISA).

*^b^* Buried area indicates the solvent-accessible surface area of monomeric units buried upon assembly formation

*^c^* Δ*G*^int^ indicates the solvation free energy gain upon formation of the assembly. Negative values of Δ*G*^int^ mean that subunits favor to from complex.

*^d^* Δ*G*^diss^ indicates the free energy of assembly dissociation. Positive values of Δ*G*^diss^ indicate that an external driving force should be applied in order to dissociate the assembly, therefore assemblies with Δ*G*^diss^ over 0 are thermodynamically stable.
