# Supplementary material for: Crystal Structure of Vaccinia Viral A27 Protein Reveals a Novel Structure Critical for Its Function and Complex Formation with A26 Protein
Source: PLoS Pathog. 2013 Aug 22;9(8):e1003563. doi: 10.1371/journal.ppat.1003563 (PMC3749956; doi:10.1371/journal.ppat.1003563)
Supplement: Table S3 — Size exclusion chromatography (SEC) of wild type and mutant tA27 proteins. (DOCX) [file ppat.1003563.s007.docx]

| **Abbreviations** | **Mutation sites** |
| --- | --- |
| tA27-WT | aa 21-84, C71A/C72A |
| tA27-TM-N | aa 21-84, L47A/L51A/L54A/C71A/C72A |
| tA27-TM-C | aa 21-84, I68A/C71A/C72A/N75A/L82A |
| tA27-6A | aa 21-84, L47A/L51A/L54A/I68A/C71A/C72A/N75A/L82A |

|  |  | **tA27-WT** | | **tA27-TM-N** | | **tA27-TM-C** | | **tA27-6A** | |
| --- | --- | --- | --- | --- | --- | --- | --- | --- | --- |
| **pH** | **Conc.** | **Elution vol.** | **M.W (kDa)** | **Elution vol.** | **M.W (kDa)** | **Elution vol.** | **M.W (kDa)** | **Elution vol.** | **M.W (kDa)** |
| 7.5 | 1mg/ml | 13.13 ml | 24.4 | 13.67 ml | 19.8 | 13.41 ml | 21.9 | 13.3 ml | 22.9 |
| 7.5 | 9.5mg/ml | 12.47 ml | 31.4 | 13.4 ml | 22 | 13.1 ml | 24.7 | 13.34 ml | 22.5 |
| 3 | 1mg/ml | 14.49 ml |  | 14.53 ml |  |  |  |  |  |
| 3 | 9. mg/ml | 13.95 ml |  | 14.77 ml |  |  |  |  |  |
